# ExaFEL: extreme-scale real-time data processing for X-ray free electron laser science

**DOI:** 10.3389/fhpcp.2024.1414569

**Published:** 2024-10-02

**Authors:** Johannes P. Blaschke, Robert Bolotovsky, Aaron S. Brewster, Jeffrey Donatelli, Antoine DuJardin, Wu-chun Feng, Vidya Ganapati, Wilko Kroeger, Derek Mendez, Peter McCorquodale, Seema Mirchandaney, Christopher P. O’Grady, Daniel W. Paley, Amedeo Perazzo, Frederic P. Poitevin, Billy K. Poon, Vinay B. Ramakrishnaiah, Nicholas K. Sauter, Niteya Shah, Elliott Slaughter, Christine Sweeney, Daniel Tchoń, Monarin Uervirojnangkoorn, Felix Wittwer, Michael E. Wall, Chun Hong Yoon, Iris D. Young

**Affiliations:** 1 National Energy Research Scientific Computing Center (NERSC), Lawrence Berkeley National Laboratory, Berkeley, CA, United States; 2 Molecular Biophysics and Integrated Bioimaging Division, Lawrence Berkeley National Laboratory, Berkeley, CA, United States; 3 Center for Advanced Mathematics for Energy Research Applications (CAMERA), Lawrence Berkeley National Laboratory, Berkeley, CA, United States; 4 SLAC National Accelerator Laboratory, Menlo Park, CA, United States; 5 Department of Computer Science, Virginia Polytechnic Institute and State University, Blacksburg, VA, United States; 6 Applied Numerical Algorithms Group (ANAG), Lawrence Berkeley National Laboratory, Berkeley, CA, United States; 7 Computer, Computational and Statistical Sciences Division, Los Alamos National Laboratory, Los Alamos, NM, United States; 8 Virginia Polytechnic Institute and State University, Blacksburg, VA, United States

**Keywords:** exascale, Single Particle Imaging, Serial Femtosecond Crystallography, hardware acceleration, data-intensive, interfacility, real-time processing, high-performance computing

## Abstract

ExaFEL is an HPC-capable X-ray Free Electron Laser (XFEL) data analysis software suite for both Serial Femtosecond Crystallography (SFX) and Single Particle Imaging (SPI) developed in collaboration with the Linac Coherent Lightsource (LCLS), Lawrence Berkeley National Laboratory (LBNL) and Los Alamos National Laboratory. ExaFEL supports real-time data analysis via a cross-facility workflow spanning LCLS and HPC centers such as NERSC and OLCF. Our work therefore constitutes initial path-finding for the US Department of Energy’s (DOE) Integrated Research Infrastructure (IRI) program. We present the ExaFEL team’s 7 years of experience in developing real-time XFEL data analysis software for the DOE’s exascale supercomputers. We present our experiences and lessons learned with the Perlmutter and Frontier supercomputers. Furthermore we outline essential data center services (and the implications for institutional policy) required for real-time data analysis. Finally we summarize our software and performance engineering approaches and our experiences with NERSC’s Perlmutter and OLCF’s Frontier systems. This work is intended to be a practical blueprint for similar efforts in integrating exascale compute resources into other cross-facility workflows.

## 1 Introduction

The Exascale Computing Project (ECP) was a large-scale project within the Department of Energy (DOE) to reach the era of exascale supercomputers, high-performance computers capable of more than 10^18^ double-precision floating-point operations per second (Messina, 2017). The goal of ECP was not only to develop and construct the hardware platforms, but also to prepare scientific software applications for these new capabilities (Evans et al., 2022). Here we describe the accomplishments as well as lessons learned while developing the application for “Data Analytics at the Exascale for Free Electron Lasers” (ExaFEL), a collaboration between SLAC National Accelerator Laboratory, Lawrence Berkeley National Laboratory, and Los Alamos National Laboratory. ExaFEL addressed the challenge of developing cross-facility exascale-capable workflows for quasi real-time analysis of experimental data from user facilities. Tackling this compute-intensive and data-intensive challenge required exceptional coordination between a diverse set of people and facilities as well as novel approaches and new software technologies.

The ExaFEL science workflow begins in a hutch at a user facility such as the Linac Coherent Light Source (LCLS), where a scientific instrument generates diffraction images. With the recent major upgrade to LCLS (LCLS-II), the light source moves from firing at 120 pulses to as many as one million pulses per second. This new capability together with recent advances in detector technologies are expected to deliver data rates at the scale of terabytes per second. These large volumes of data are handled by LCLS Data Systems (Thayer et al., 2017), where streams of data from different detectors are assembled into a format readily available for users through Python interfaces. Next, the data are sent over a fast network (e.g., ESNet^1^) to a high-performance computing facility such as NERSC, OLCF or ALCF, where they are promptly analyzed on a supercomputer. The loop is closed by sending the results back to the experimental site in real time, where scientists observe and supervise every step of the data collection and reconstruction.

LCLS is an X-ray free-electron laser (XFEL) that produces ultra-bright and ultra-short X-ray pulses. The pulses can be used to study the structure of molecules, to investigate fast phenomena, or to probe extreme energy scales. Due to these unique capabilities, experimental time at XFELs is in high demand, and only a small selection of requests can be met. It is therefore critical to make the best use of the scarce experimental time through fast feedback. Real-time feedback minimizes the time between measurement and first scientific results. Crucially, it allows the experiment’s operators to quickly decide how to proceed once a dataset is complete, whether or not to continue with the next sample, or if the experimental setup needs to be adjusted.

XFEL experiments are generally high-stakes, collaborative efforts, which limits their reproducibility in a second experiment. In the past, real-time feedback was typically not available and the data quality was not assessed until after the experiment was over. The steering of the experiment had to err on the safe side since a subsequent experiment would typically not be possible for several months, if ever. This meant scarce and expensive experimental time was not used efficiently, reducing the total potential science output of the facility.

In the following Section 1.1, we will introduce the science drivers that led to the development of the ExaFEL project, and in Section 1.2 we will introduce the unique experimental facility exascale data streaming requirements met by the ExaFEL project.

### 1.1 Science drivers

**FIGURE 1:**
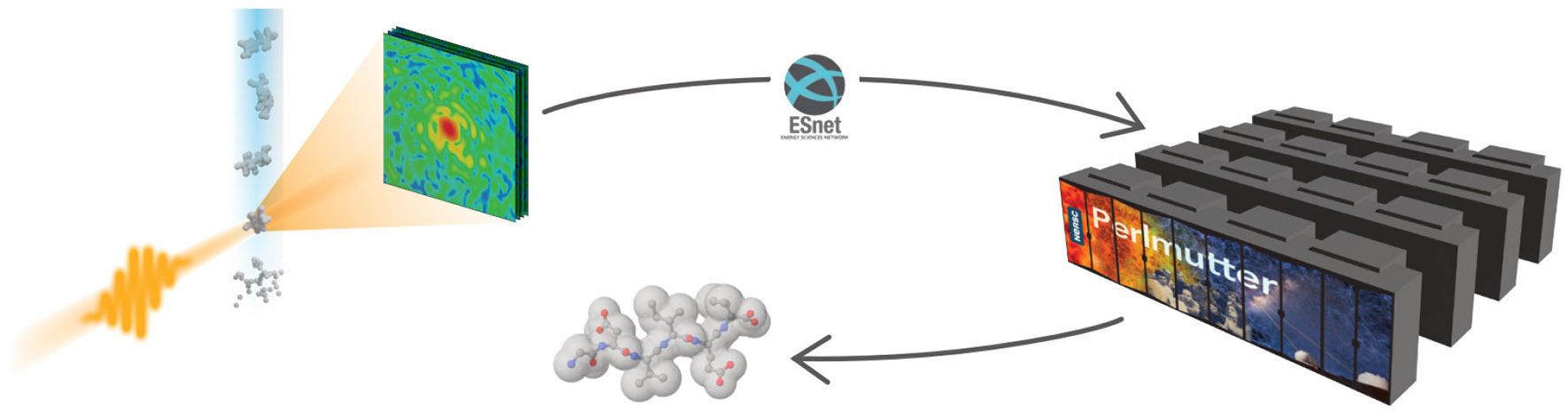
The XFEL diffraction workflow. A water jet carrying particles or molecules is shot into the X-ray beam. The scattered X-rays are recorded on a detector in the form of diffraction patterns, which are sent to a computing facility for analysis. The results are then sent back to the experiment.

Two of the most important science drivers at LCLS are Serial Femtosecond Crystallography (SFX or nanocrystallography) and Single Particle Imaging (SPI). In these methods, a stream of nearly identical microscopic samples is shot into the focus of the X-ray beam. When the beam hits a sample, the X-rays are scattered/diffracted and the diffraction pattern is recorded with an image detector. From the distribution of the diffracted X-rays, the molecular structure of the sample can be reconstructed. Reconstructing the full 3D information of the sample requires hundreds to thousands of diffraction patterns to cover all sample orientations. Because the intensity of the X-ray pulse destroys every sample it hits, each sample can only generate one diffraction pattern. For a full dataset, patterns from many samples must be combined correctly, but the orientation of each sample is random and unknown. The orientation must be derived computationally by referencing the diffraction patterns against each other. Furthermore, the samples are not perfect copies of each other, requiring additional modeling and complicating cross referencing. The typical workflow is illustrated in Figure 1.

About one-third of X-ray beam time allocations at LCLS are currently awarded to SFX experiments (Keedy et al., 2015; Ayyer et al., 2016). SFX experiments offer huge benefits to the study of biological macromolecules, including the availability of femtosecond time resolution and the avoidance of radiation damage under physiological conditions. LCLS makes it possible to probe molecular dynamics in the sub-picosecond time domain by triggering chemical changes with an optical pump/X-ray probe arrangement (Pande et al., 2016).

The high X-ray pulse rate and ultra-high brightness of LCLS make it possible to determine the structure of individual molecules with SPI, mapping out their natural variation in conformation and flexibility. Structural heterogeneities, such as changes in the size and shape of nanoparticles or conformational flexibility in macromolecules, are at the basis of understanding, predicting, and eventually engineering functional properties in biological, material, and energy sciences. In pursuit of these goals, the classification of diffraction patterns into conformational states and the subsequent phasing of the data to yield 3D electron density are computational challenges. New algorithms, such as the Multi-Tiered Iterative Phasing (M-TIP) (Donatelli et al., 2017) approach (Section 2.3), are changing the way we understand how to process these data.

### 1.2 The exascale streaming problem

The LCLS-II upgrade increased the maximum repetition rate of the machine from 120 to 1m (Abbamonte et al., 2015). The throughput from the front-end electronics is expected to increase by three orders of magnitude by virtue of the increase in detector readout rates from 120 Hz to 50 kHz as well as the new ability to operate multiple beam-lines concurrently. On the data analytics side, compute-intensive algorithms are necessary to probe atomic features with the highest spatial resolution. This combination of high data rates, compute-intensive data analysis and the need for quick feedback pushes SFX and SPI into the exascale regime.

For SFX, the nature of the crystallography experiment requires that data be collected under a series of variable conditions, e.g., pump-probe protocols involving differing excitation energies, reactions being followed under a series of time delays, or specimens being prepared under differing The experimental time necessary to investigate a single experimental condition is on the order of 10 min. We have shown in Lyubimov et al. (2016) that a more accurate analysis than our current best practices is possible with protocols needing a thousand times more CPU time.

For SPI, with the projected image acquisition rate of 50 and an estimated sample hit rate of 10, LCLS-II is projected to produce useful data throughput on the order of terabits per second. In order to obtain a high-resolution reconstruction for a sample with a large number of conformational states using the estimated image count, the pre-ExaFEL M-TIP workflow would require more than 100 compute hours. The availability of exascale computing resources and a high-performance computing (HPC) workflow that can handle incremental bursts of data will enable data analysis to be performed on the fly, providing immediate feedback on the quality of the experimental data, while determining the 3D structure of the sample at the same time.

The remainder of this paper is structured as follows: Section 2 describes the three main software packages developed by ExaFEL. Section 3 describes the unique challenge faced by the ExaFEL team running an exascale computing workflow across facilities in real time. Section 3.1 discusses ExaFEL’s experiences and path-finding work that is expected to be of value for the DOE’s Integrated Research Infrastructure (IRI) Program (Miller et al., 2023). Section 3.2 discusses best practices for this infrastructure. Section 4 discusses the lessons learned developing the ExaFEL software, and Section 5 concludes the paper. Section 1 of the Supplementary material contains information on the Computational Crystallography Toolbox and FFT optimizations.

## 2 Design and execution of HPC software technologies

**FIGURE 2:**
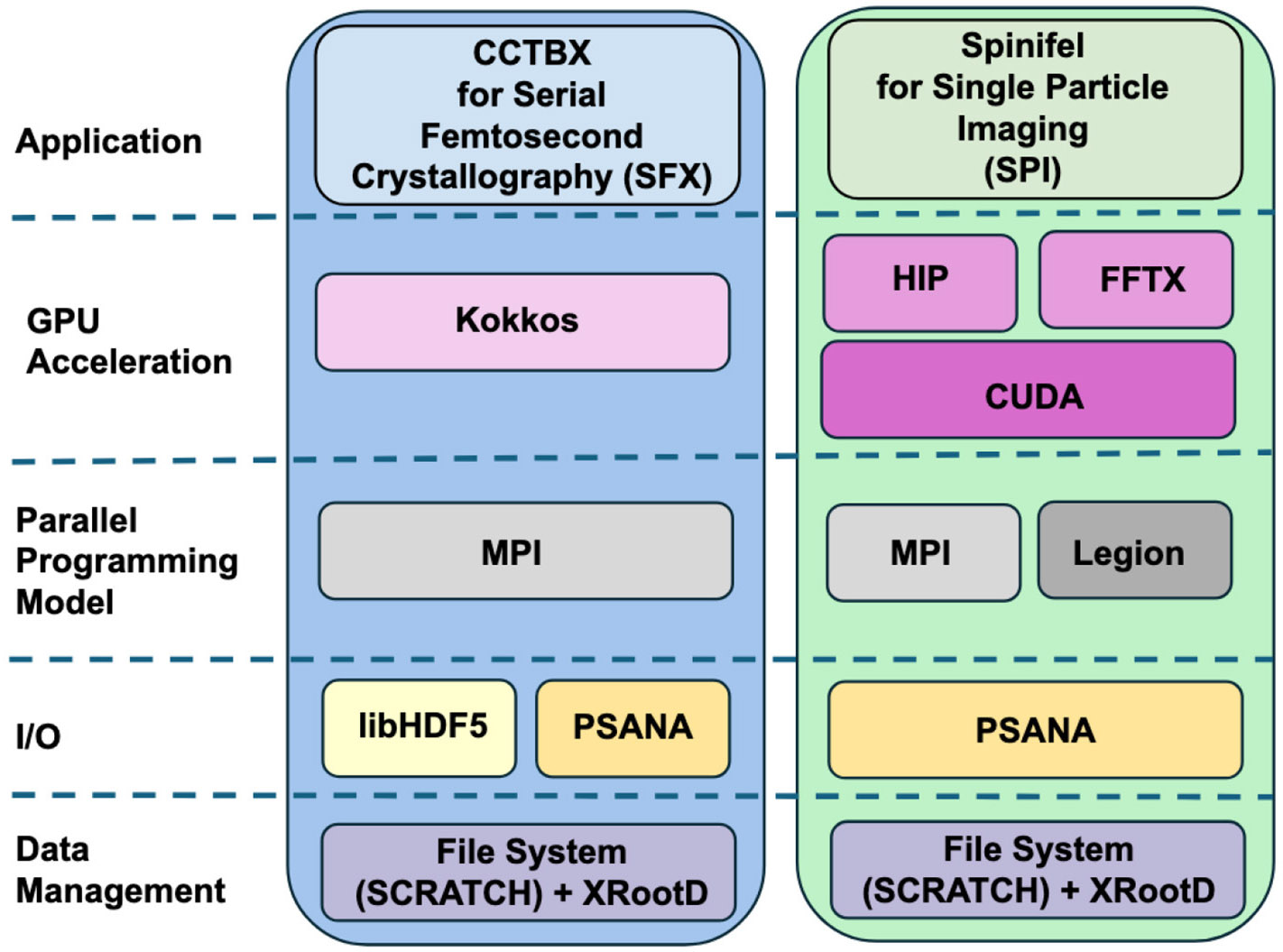
The ExaFEL software stack. Data processing applications *cctbx* and *Spinifel* take different approaches for GPU acceleration, but both use *psana*, MPI, and file system plus *XRootD* for data management, with *Spinifel* alternatively using the Legion parallel programming model and *cctbx* alternatively using *libHDF5* for I/O.

ExaFEL’s software development centered around packages for data handling at LCLS (*psana*), and the analysis of SFX (*cctbx*) and SPI (*Spinifel*) data. We give a brief overview of these software products and show how their computational motifs helped to support exascale and cross-facility real-time computing. Figure 2 provides a high-level view of the software components used.

### 2.1 LCLS data systems: PSANA

**FIGURE 3:**
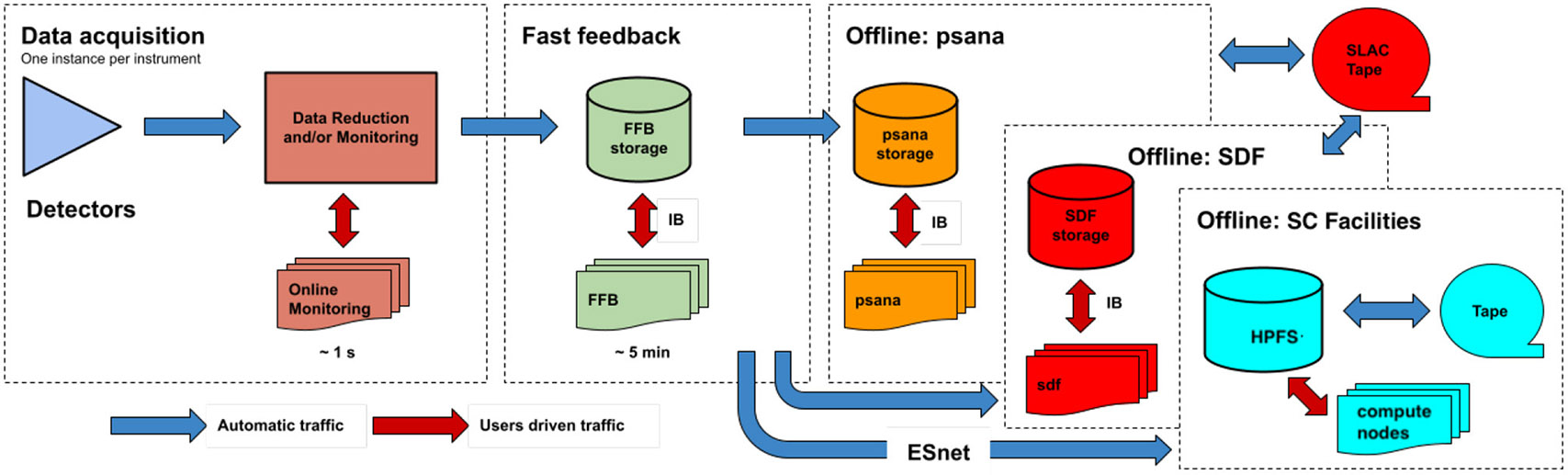
LCLS data flow indicating different stages where data analysis can be performed. Online monitoring is the lowest-latency option, wherein data are made available for analysis even before they are written to file, only about a second after raw signals are recorded on the various detectors. The next fastest alternative is access to data files in fast feedback (FFB) temporary storage. From FFB, files are transferred to either *psana* or Scientific Data Facility (SDF) computing systems for medium-term storage and “offline” analysis, and optionally to external supercomputing facilities as well using ESnet. Data stored on *psana*, SDF, or the high performance file systems (HPFS) at NERSC and OLCF are later automatically backed up to tape for long-term storage. Data transfer takes place over regular Ethernet networking, or via InfiniBand (IB).

The data processing framework at LCLS (Figure 3) begins at data acquisition time where data from different detectors are filtered and monitored prior to being written to fast-feedback storage. This storage allows users to perform analyses while the data are being written. For larger data volumes, LCLS can optionally stream the data to other computing clusters or supercomputing clusters (via ESnet) to facilitate real-time feedback. Unlike the previous LCLS-I data acquisition system, LCLS-II data acquisition system writes out separate files for each detector: one file with the large-volume “big data”, and an additional supporting “small data” file that includes *fseek* offsets and sizes for each X-ray shot. Both files are written in a lightweight custom format called “xtc2”.

**FIGURE 4:**
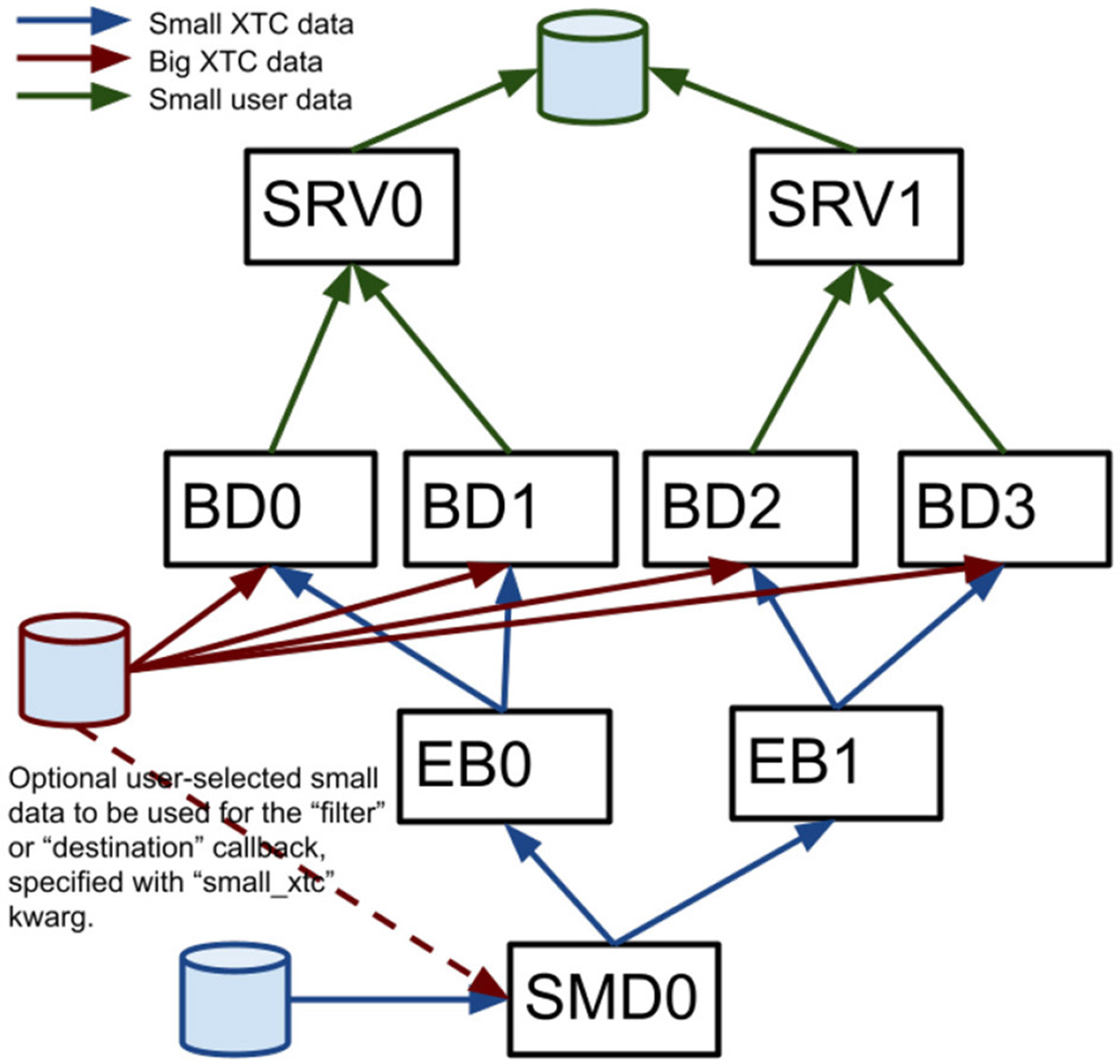
A diagram shows the four MPI layers for LCLS-II offline data management. Boxes represent MPI processes for SMall Data rank 0 (SMD0), “Event Builder” (EB), “Big Data” (BD) and “Server” (SRV).

Most data analysis software run at LCLS (including *cctbx* and *Spinifel*) relies on the *psana* software framework to read measured data. With the recent LCLS-II upgrade, the offline data analysis package “PSANA-II” is used with the newer xtc2 format data. An older “PSANA-I” software framework for LCLS-I data is supported, but is not used for the large-scale ExaFEL analysis discussed here. PSANA-II uses Message Passing Interface (MPI) (Gropp et al., 1996) to manage scalable data reading and analysis. The two types of data files written by the data acquisition system described previously are used to enable this by distributing all detector data from different X-ray shots to different cores. Four MPI-communication layers (Figure 4) work together starting from the “SMall Data rank 0” (SMD0) process, which runs as a multi-threaded single process. This process reads all SmallData xtc2 files in chunks and associates the *fseek* offsets for all detectors that belong to the same X-ray shot using the recorded timing-system timestamps. This process also optionally includes user-specified larger xtc2 data to be used for X-ray shot filtering. These chunks with *fseek* offsets and user filtering data are distributed to “Event Builder” (EB) cores where the small data are first made available to user Python code for filtering purposes, giving the user the ability to reject events that they are not interested in, thus avoiding needless big-data fetch time. The events that pass this filter stage are passed on to the “Big Data” (BD) cores which use the *fseek* offsets and sizes to read the big detector data and process it with user-written Python code. Reduced-size processed data can then be passed to the “Server” (SRV) cores for real-time monitoring or persisting to Hierarchical Data Format version 5 (HDF5) files. All communication layers apart from SMD0 can be scaled to arbitrary numbers of nodes/cores using MPI.

**FIGURE 5:**
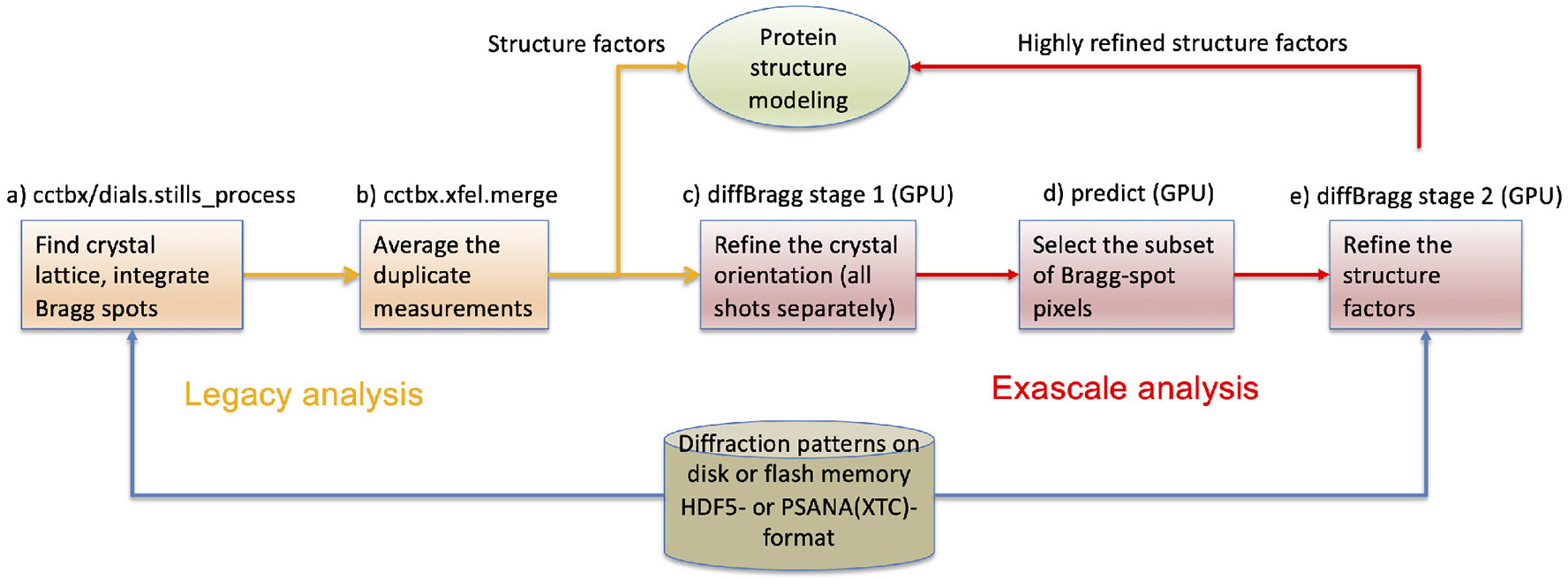
The protein crystallography workflow with data reduction software components (a–e) identified. Legacy analysis (a, b) terminates with the estimation of structure factors that serve as Fourier coefficients to compute an electron density map and ultimately the atomic structure. Exascale analysis (c–e) carries further with the goal of estimating the structure factors more accurately. It assumes that steps (a, b) have been completed, thus the legacy analysis is always run first. The raw data remain on disk for both analyses.

**FIGURE 6:**
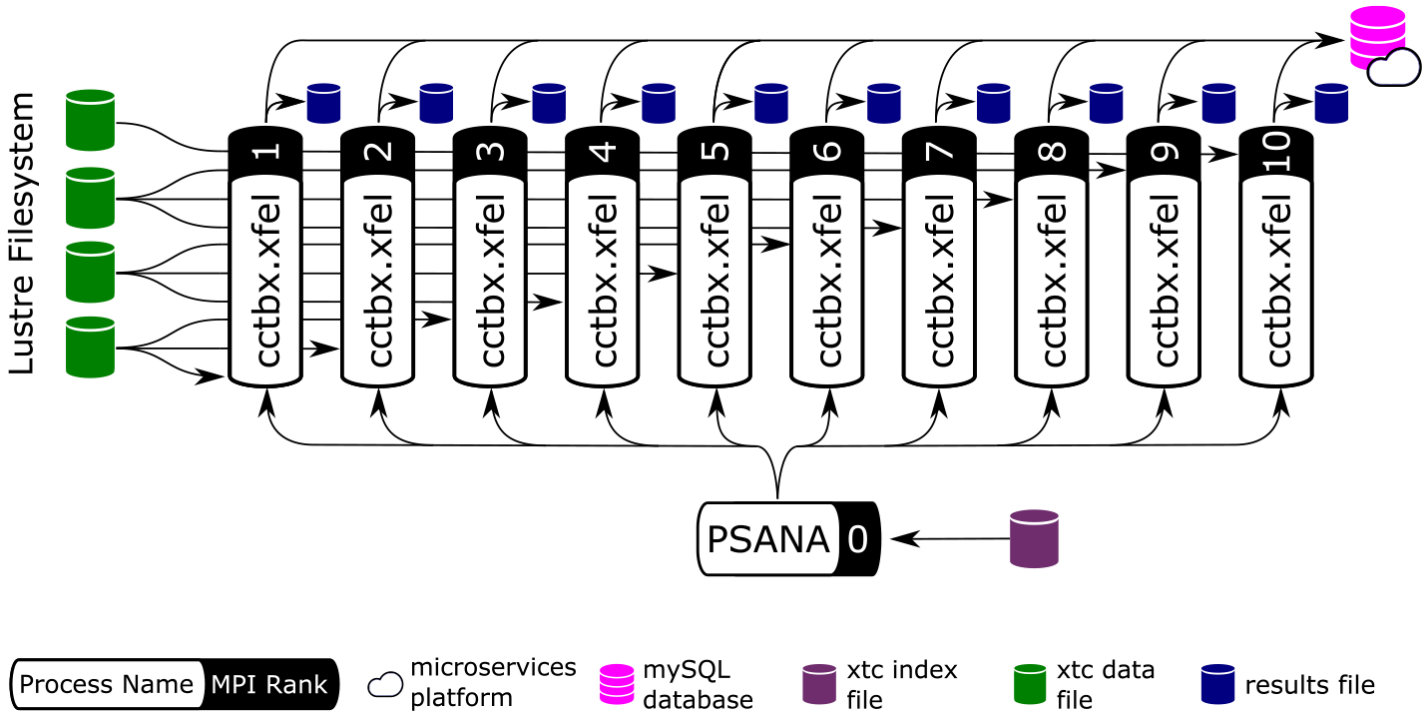
Producer-consumer structure of *cctbx* analysis workers. Arrows represent data-flow. *cctbx* relies on MPI parallelism to distribute work across ranks. We employ a producer/consumer model to distribute work and achieve load balancing. Data are provided by *psana* (*cf.* Section 2.1), which runs on the first MPI rank (rank 0). *psana* reads an index file and distributes work to the *cctbx* workers. The resulting program is a flat tree of MPI ranks with data analysis ranks located at leaves. Workers access data directly by reading the raw data files using offsets provided by the “PSANA” (root) tree node. MPI collective communication (MPI_Bcast, MPI_Allgather, MPI_Allreduce) are used to share data between MPI ranks. Finally, the *cctbx* workers save their results to disk (local to each MPI rank) and report the analysis progress to a MySQL database hosted on NERSC’s Spin micro-services platform (*cf.* Section 3.1). Image adapted from Blaschke et al. (2024) with the permission of the authors.

This framework was heavily used at the beginning of the ExaFEL project. Starting in 2016 the PSANA-II software was integrated into *cctbx* (more details in Section 2.2 and Figures 5, 6) to analyze X-ray diffraction images in real time. Data were transferred to NERSC’s “Cori” supercomputer through ESNet, read and assembled with PSANA-II, and processed with *cctbx*. A data volume of 1 million images would typically take days to process on a normal computer, but we were able to analyze it in less than 7 min (or 2 kHz) when utilizing 340,000 Cori cores.

PSANA-II was also integrated with the Legion Programming System (Michael et al., 2012) to allow use of M-TIP algorithms for SPI data processing (*Spinifel*) using an asynchronous task-based model. The four layers shown in Figure 4 are considered as *tasks* in the Legion programming system. These tasks share data collections through *privileges* that allow the Legion core system to determine scheduling patterns automatically to match available computing resources. A Python interface to Legion, Pygion (Slaughter and Aiken, 2019), was also developed to provide easier accessibility to HPC for scientific users.

### 2.2 The computational crystallography toolbox: CCBTX

The computational crystallographic toolbox (CCTBX) was introduced over 20 years ago as a collaborative open-source software library. It has since grown to encompass several additional software projects and has been deployed at beamline endstations and HPC facilities around the world. A common design feature of all CCTBX-related software packages is that the top-level workflow is developed in Python, and computationally intensive tasks are developed using C++, including Kokkos (Trott et al., 2022) for portable GPU-enabled software. The interface between Python and C++ is made using *Boost.Python*.^2^

Four software packages relevant to the treatment of XFEL crystal diffraction in the ExaFEL project fall broadly under the CCTBX umbrella: (i) the *cctbx* software suite provides a foundation of core algorithms (Grosse-Kunstleve et al., 2002); (ii) *DIALS* builds on *cctbx* and provides open source implementation of specialized data reduction algorithms, as well as data readers for a vast range of file formats common to crystallography (Winter et al., 2018); (iii) the *cctbx.xfel* toolkit (Sauter et al., 2013) covers algorithms for high data rate XFEL processing using MPI (Brewster et al., 2019); and (iv) *diffBragg* extends *cctbx.xfel* and leverages modern GPUs to implement cutting-edge XFEL data analysis (Mendez et al., 2020).

Figure 5 shows how these software packages fit together with *psana* to form an exascale workflow.

#### 2.2.1 Computational motif of CCTBX

The X-ray diffraction pattern of a protein crystal samples the Fourier transform of the unit cell contents, creating a 3D array of spots, or Bragg reflections. The goal of a crystallographic experiment is to measure these peaks so the inverse Fourier transform can recover the contents of the unit cell, including atomic coordinates.

SFX diffraction patterns are wholly independent from each other, as they are each single images collected from different crystals, randomly oriented. Each pattern represents a 2D slice through the 3D space of Fourier coefficients, containing partial information about the complete dataset. The initial data reduction steps can be performed for each diffraction pattern independently (Figure 5a), thus taking advantage of massive supercomputing parallelism at the CPU level. These initial steps are documented in Section 1 of the Supplementary material.

Figure 6 shows the overall orchestration of the work, with these steps being executed for every diffraction pattern as it is read from disk. MPI divides the job into two parts. The communicator on rank 0 is used to find a pointer to the data in each image (it does not actually read the big data), and then the remainder of the work is delegated to the higher-numbered ranks for parallel processing. Each rank loads and processes a subset of all the data independently. Common analysis parameters are shared across all workers using MPI collectives (using MPI_Bcast). Results are likewise stored independently, committed to a *MySQL* database, and updated across all workers using using MPI collectives (depending on situation: MPI_Allgather or MPI_Allreduce). For any iterative algorithms (steps b and e in Figure 5), MPI collective communication occurs at both the start and the end of each iteration.

Following this work in *dials.stills_process* (Figure 5a), the measurements from all patterns need to be merged together, which is a global operation, performed by the program *cctbx.xfel.merge* (Figure 5b). Merging includes error calibration, since the integration error will not have accounted for the full set of systematic and random errors in the experiment, followed by averaging of all measured intensities mapped to the same structure factors. MPI parallelism is used here as well, but in a less obvious way. The code transitions from assigning individual MPI tasks to separate diffraction patterns to assigning them to separate structure factors; this is done with the MPI primitive all_to_all(). Statistics are computed and logged throughout this process, both in text files and in the *MySQL* XFEL GUI database, which is used for data visualization and evaluation. The CPU-based merging step has been tested up to 5 × 10^5^ diffraction patterns.

These two steps of “legacy analysis” (Figure 5a, b) were early work products of the ExaFEL project, initially put into production on the NERSC Edison and Cori systems (Blaschke et al., 2024) and since ported to Perlmutter. In contrast, the *diffBragg* program is still experimental work. With respect to parallelism, the first two steps, *diffBragg stage 1* and *predict* (Figure 5c, d), are similar to *dials.still_process* in that they perform processing steps independently for each diffraction pattern. Therefore they are again expressed as Python scripts running with MPI, with individual patterns delegated to MPI ranks for processing. Architecturally, however, they differ from the legacy steps by adding GPU processing for every image, using a custom-written kernel implemented in Kokkos (Wittwer et al., 2023) to calculate diffraction from physical principles. The kernel executes a parallel_for loop where each pass through the loop produces the total number of photons incident on one pixel, with all pixels treated independently. Our Kokkos code is portable and can be compiled on NVIDIA, AMD and Intel GPU architectures.

The final step (Figure 5e) is *diffBragg stage 2*. Whereas *stage 1* refines local parameters different for each diffraction pattern, *stage 2* refines a set of global physics parameters, namely the structure factors that are repeatedly but imprecisely measured on many separate images throughout the dataset. *Stage 2* improves the structure factor estimates with the quasi-Newton solver LBFGS (Liu and Nocedal, 1989). This is accomplished by the iterative minimization of a target function, the log-likelihood of the entire dataset given the parameters, which is a simple summation of the log-likelihood of each image. Load balancing is accomplished at program launch, where images with variable Bragg reflection counts are distributed evenly across MPI ranks using longest-processing-time-first scheduling. At the start of each iteration, each MPI rank calculates image likelihoods and their gradients with respect to each global parameter. Then, the per-rank likelihood and gradient terms are aggregated in an all-to-all reduction. The reduced terms are then broadcast back to every rank (completing a so-called MPI All-Reduce operation). Each iteration completes with the same LBFGS parameter update occurring in parallel on all MPI ranks. Repeated cycles in this manner eventually lead to global parameter convergence. We tested this GPU-based optimization step on up to 1.25 × 10^5^ diffraction patterns on NERSC Perlmutter and OLCF Frontier.

#### 2.2.2 Developing portable GPU-accelerated kernels using Kokkos

*diffBragg* benefits significantly from GPU acceleration. To assess potential benefits and compare different portability options, we created *nanoBragg*, a stand-alone program of the simulation core of *diffBragg*, which we then ported to CUDA and Kokkos (Mendez et al., 2020; Sauter et al., 2020; Wittwer et al., 2023). *nanoBragg* uses X-ray tracing to simulate the diffraction signal of each individual detector pixel, a highly parallelizable task well suited to GPUs. The initial code followed the paradigm of using mainly Python code with critical sections written in C++. These critical sections were first ported to CUDA to assess the GPU performance. The results are summarized in Table 1, with Edison and Cori using the C++ version. The CUDA version shows a dramatic speed-up, reducing the time to simulate 100,000 images from hours to minutes. Accordingly, a CUDA version was also written for *diffBragg*, showing similar speed-ups. However, Frontier and Aurora, the Exascale systems at OLCF and ALCF, do not support CUDA and require additional code porting. For *nanoBragg* and *diffBragg*, we had two options: Either keep the CUDA version and write dedicated implementations for each vendor platform, or rewrite it using a portability layer such as OpenACC or Kokkos. We chose the second option because it requires writing and maintaining only a single code-base.

We used the CUDA implementation as the starting point for the Kokkos port, which allowed us to reuse large parts of the data transfer and kernel structure. Most of the work was straightforward, replacing CUDA arrays with Kokkos views and converting CUDA kernels into Kokkos execution patterns. However, there were also a number of challenges.

One hurdle was that Kokkos requires a call to its initializer before any other Kokkos code is used and similarly requires a call to the Kokkos finalizer after all other Kokkos code is deconstructed. In C++ this can be achieved via code regions, as the regions limit the lifetime of all variables and objects within it. In Python, this is more complicated, as the garbage collector decides when objects are actually deleted. To manage this, some scripts needed a dedicated clean-up step so that all Kokkos objects and functions were destroyed before the Kokkos finalizer is called.

Another hurdle was the use of certain third-party libraries in *diffBragg*. CUDA is widely supported by many libraries that are not directly connected to CUDA or NVIDIA. One of these is the *Eigen* library, which is used in the C++ and CUDA version of *diffBragg* for basic linear algebra operations. However, *Eigen* does not support Kokkos. We had to construct Kokkos implementations of all necessary algebra operations.

Lastly, we faced pitfalls as novice Kokkos adopters. Among these was the difficulty of using execution patterns in class methods, unexpected performance differences between different hardware platforms, and the general challenge of profiling Kokkos applications. Because Kokkos objects are heavily templated, the generated function and object names are very long and complex. It is somewhat difficult to cross-reference the profiler output to the source code. While some programs, such as Nsight systems, have the option to rename function and objects, many others only show the full name.

#### 2.2.3 Low-level engineering of kernel performance

**FIGURE 7:**
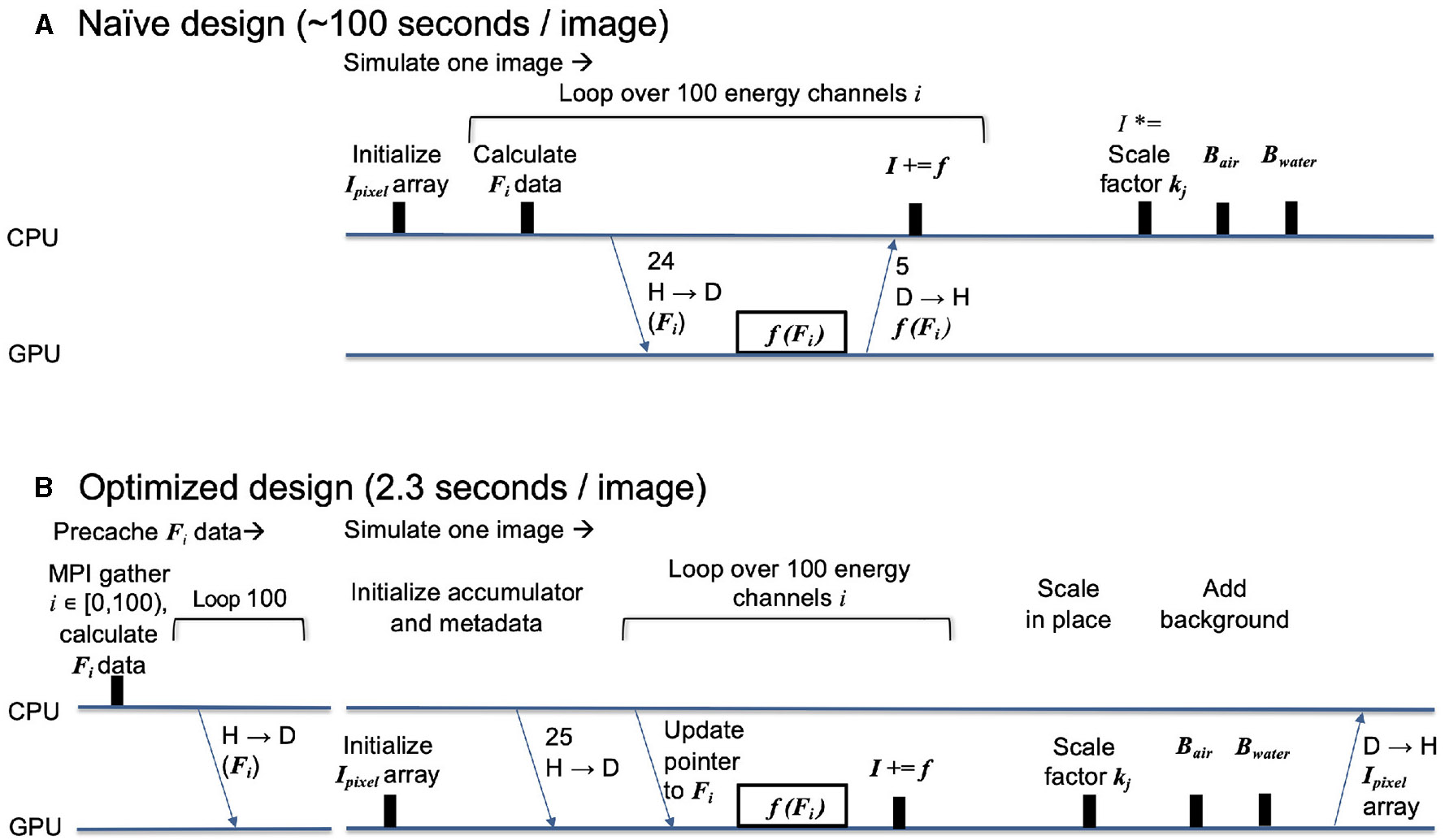
Time progression diagrams showing performance engineering in the *nanoBragg* kernel (initially written in CUDA, then ported to Kokkos). The timelines show interaction between host (H, CPU) and device (D, GPU) for the naïve design **(A)** organized around a single kernel and for the optimized redesign **(B)** using the same core kernel, but adding auxiliary data structures and kernels for summation, scaling, and background calculation, so that essentially all calculation is done on GPU. Inputs consist of the structure factor array *F_i_* for energy channel *i*, with one array for each channel, along with several metadata arrays. The output consists of the final pixel intensity array *I*_pixel_, having a summed contribution *f*(*F*_*i*_) from each energy channel, an overall multiplicative scaling factor *k_j_*, plus air and water scattering *B*_air_ and *B*_water_.

We used *nanoBragg* (Sauter et al., 2020) as a testbed for creating an efficient GPU-centered workflow. A naïve first approach is illustrated in Figure 7A, where we sum up, on the GPU, contributions of structure factors to each pixel in the output image, taken over 100 individual energy channels. A benefit of our object-oriented approach is that the entire interaction between CPU host and GPU device is encapsulated in a method of a Python class, so that it is easy to produce alternate workflows by Python rescripting. Indeed, the loop over energy channels is performed at the Python level.

However, a weakness in our initial design is that each energy channel iteration includes a time-consuming host-to-device data transfer of the structure factors prior to kernel execution, and then a device-to-host transfer of the incremental pixel intensities to be added to the final output array, which stays on the CPU. This is especially wasteful as the MPI rank repeatedly transfers the same structure factors from host to device as it performs potentially thousands of image simulations with slightly different metadata. Therefore, in our redesign, Figure 7B, all the structure factor arrays (one for each energy channel) are transferred to high-bandwidth device memory upon initialization, so the data can be repeatedly used to simulate many images. Each MPI rank still maintains its own arrays on-device, i.e., we do not share pointers among ranks, although there might be potential savings by making memory global over all devices and nodes.

The redesign also requires that we initialize the output array on the device instead of the host, and then keep it there until all the contributions (and multiplicative scale factors) are applied. The resulting design is 40-fold more efficient as it minimizes data transfer and completely eliminates CPU-based array math.

#### 2.2.4 CCTBX performance scaling

A main challenge of the ExaFEL project was to prepare for a near-future scenario where it may be possible to collect useful diffraction patterns at rates up to 5,000 Hzin contrast to the current 120 Hz. At the same time, we wish to apply our most advanced data reduction algorithm, *diffBragg*, to resolve tiny differences in the atomic structure as a function of experimental variables. These variables include the pump-probe delay time, allowing us to examine the time progression of enzymatic reactions, as already demonstrated for systems such as photosystem II (Bhowmick et al., 2023). As *diffBragg* is still work in progress, we assess here the limits of computation using the resources available today on Frontier.

**FIGURE 8:**
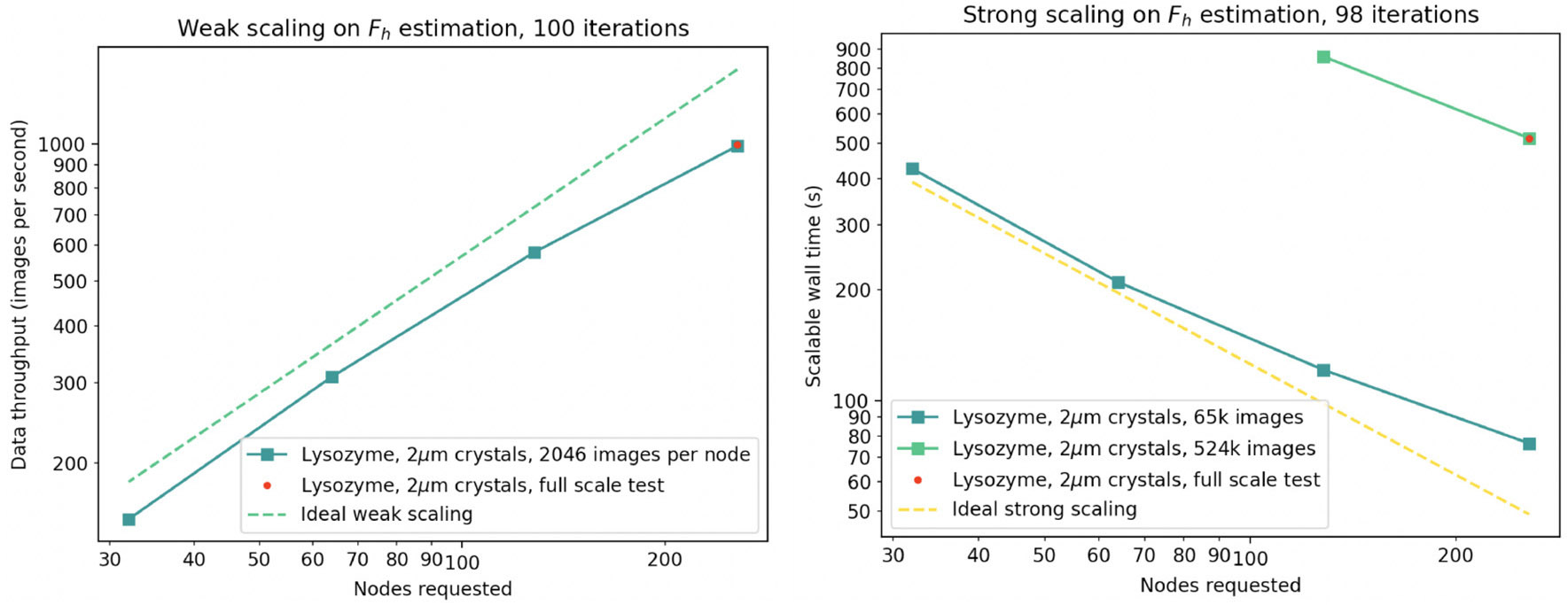
Demonstration of weak **(left)** and strong **(right)** scaling of *diffBragg stage 2* on up to 256 Frontier nodes. The red dot indicates the execution of the same tranche during simultaneous execution of 20 tranches on 5,120 nodes.

We assumed that one “dataset” representing a single time point in an enzymatic progression would consist of up to 500k diffraction patterns (2^19^), about 10-fold larger than present-day experiments, thus serving as a useful upper limit. We found that for one dataset, 256 Frontier nodes allow a sufficiently fast calculation when the data are delegated to 4,096 MPI ranks (16 ranks per node, or 2 ranks per Graphic Compute Die) as shown in Figure 8. Each node is equipped with 512 GB DDR4 RAM, and so each MPI rank is allowed 32 GB CPU memory to hold 128 raw diffraction patterns plus all intermediate calculations. For comparison, the raw data assigned to each rank (128 images, each 16 Megapixel with 2-byte depth), are about 2.2 GB compressed.

We executed numerous trials to determine if *diffBragg* could be used to reduce data quickly enough to provide feedback to the experiment. In our hypothetical scenario the 2^19^ experimental images would be acquired in about 100 s, and our data reduction trials indicate that hundreds of iterative cycles of parameter estimation can yield a result in 30 to 120 min. In each cycle we calculate the diffraction pattern and its first derivative from the current estimated parameters, all on GPU. These results are returned to CPU and used to form gradients needed for the LBFGS quasi-Newton method, which generates better parameter estimates for the next iteration. The LBFGS step, on CPU, is the gathering point where results from all MPI ranks are merged together.

With the single-dataset workflow in place, we then investigated how to stack the analysis of many data sets at once, as would be necessary during a real experiment. Our preferred plan was to preserve the model of one-*Slurm*-job-per-dataset, which is currently implemented at Perlmutter and described in Blaschke et al. (2024). By arrangement with NERSC, we reserve a fixed number of supercomputer nodes for the duration of the data collection shift. An external workflow manager (our interactive GUI of Section 4.1) then submits repeated *Slurm* jobs to the reserved queue every few minutes, each time new data become available. The results provide quick experimental feedback to guide decision making.

The reservation paradigm was not available for a full-scale test at Frontier. Instead, we wrote a single *Slurm* script to encapsulate a compound job, analyzing 20 datasets simultaneously on 5120 nodes, using parallel srun commands to start separate MPI communicators. While differing from our proposed workflow in the job initiation step, the single-*Slurm*-job workflow reproduced most aspects of inter- and intra-node communication, file I/O, memory usage, and raw computing power. Our main result is that under the fastest data collection rate anticipated in the future (10^7^ diffraction patterns collected in 33 min), our groundbreaking algorithm for estimating structure factors, *diffBragg stage 2*, can be run on roughly the same time scale as data collection. Even moderately large problem sizes yield a meaningful number of structure factor estimations (and by extension, a biologically meaningful quantitative difference between related molecular structures) in two hours.

### 2.3 Software for Single Particle Imaging: *Spinifel*

With LCLS-II upgrades, single-particle diffraction experiments are expected to operate at rates between 100 and 1,000 kHz and produce 20-Megapixel images. Furthermore, there is a desire for experimentalists to obtain real-time feedback from these experiments, which, when combined with the expected imaging rates, will require a vast amount of computing power. In order to meet these needs, we designed and developed *Spinifel*, a software package for determining 3D molecular structure from an ensemble of single-particle diffraction patterns at scale. Similar to *cctbx* (Section 2.2), *Spinifel*’s top-level workflow is developed in Python with computationally intensive tasks offloaded to compiled Python extension modules written in C++, HIP, and CUDA. The interface between Python and C++ is made using *Pybind11*.^3^ Unlike *cctbx*, the extension modules are not portable between NVIDIA and AMD. The decision of which code to call is made by the Python workflow at runtime.

*Spinifel* is based on the Single-Particle Multi-Tiered Iterative Phasing (SPMTIP) algorithm introduced in Donatelli et al. (2017). In particular, SPMTIP simultaneously determines the conformational states, orientations, 3D intensity complex phases, and the underlying structure in a single iterative framework. By simultaneously determining all missing degrees of freedom in this manner, SPMTIP is able to leverage constraints on the possible 3D structures, e.g., sparsity, positive density, symmetry, density statistics, etc., to boost the effective information content of the system and reduce the amount of data needed to obtain a successful reconstruction.

As part of the ExaFEL project, *Spinifel* was built by redesigning some of the core computational routines in SPMTIP to improve computational complexity for large data sets and implementing these routines to run and scale on next-generation exascale computer architectures. In particular, the SPMTIP routines in *Spinifel* were converted from a spherical-polar-grid framework to a Cartesian grid/sparse data framework based on non-uniform Fast Fourier transforms, which reduces the computational complexity of the algorithm from *O*(*N*^4^*D*) to *O*(*N*^2^*D*), where *N* is the number of Shannon channels (i.e., grid points per dimension) modeled in the reconstruction and *D* is the number of diffraction patterns used. Furthermore, we scaled *Spinifel* code to run efficiently on Frontier with additional functionality to reconstruct multiple particle conformations.

#### 2.3.1 Computational motif of *Spinifel*

**FIGURE 9:**
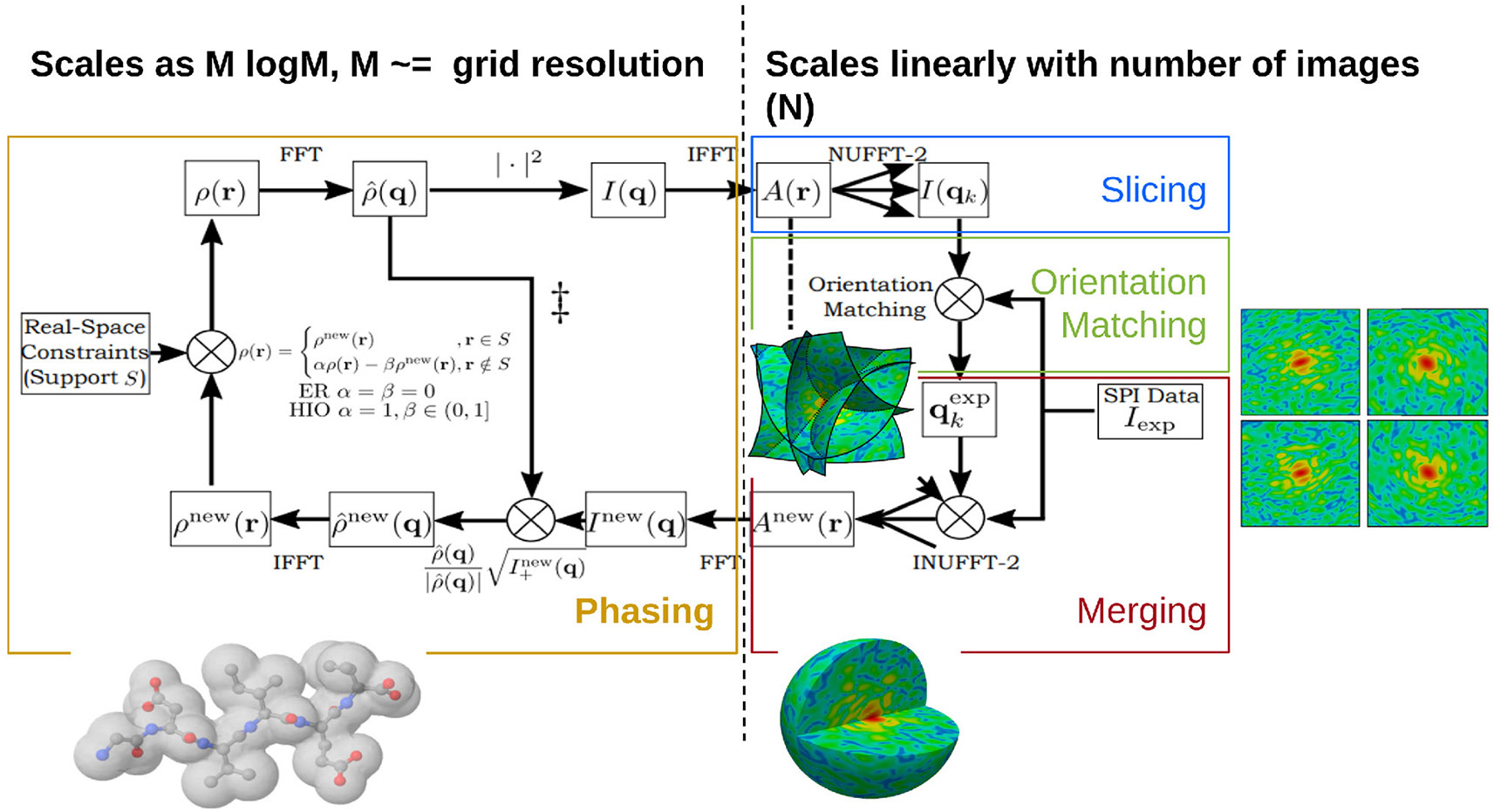
Scalable SPMTIP algorithm realized by *Spinifel* code for single particle imaging reconstruction using a Cartesian basis. The portion on the left side (Phasing: yellow box) indicates code that scales as *O*(*Mlog*(*M*)) with assigned grid resolution *M*, while on the right side: Slicing (blue), Orientation Matching (green), and Merging (red) indicate parallel code and GPU Offloading that scales as *O*(*N*) with number of images *N*. The targets for GPU offloading are implemented for the forward transform, orientation matching, and adjoint transform, respectively. Image adapted from Chang et al. (2021) with the permission of the authors.

Figure 9 shows how the computations of *Spinifel* can be decomposed into three subproblems:

1. “Slicing” and “Orientation Matching” recover the orientations corresponding to each of the diffraction patterns.
2. “Merging” aggregates the oriented diffraction patterns into a single diffraction volume, which is equivalent to reconstructing the autocorrelation of the electron density of the particle.
3. “Phasing” recovers the missing phase information of the diffraction volume, which is equivalent to reconstructing the electron density of the particle.

The solution to these three subproblems is scaled in *Spinifel*, as described below. In particular, for our problem, the experimental data are very large (10^12^-10^15^ floats) and need to be distributed over multiple nodes, but the molecular density model that we wish to reconstruct and most of the intermediate quantities (100^3^-500^3^ floats) will typically fit on one or a few nodes.

Slicing and orientation matching are solved by first generating a set of 2D reference images on a predetermined selection of orientations (given as a 3D grid of Euler angles), by computing the Non-Uniform Fast Fourier Transform (NUFFT) of the autocorrelation of the current electron density estimate of the molecular model. Next, each experimental 2D diffraction image is compared against all of the reference images. The orientation of the experimental image is then selected as the one that minimizes the distance (*e.g*., a weighted L2 norm or negative log-likelihood) between the experimental and corresponding reference images. This process was scaled over multiple nodes by providing each node with a copy of the reference images (involving a single initial scattering operation) and distributing the experimental images over the nodes; these images are kept in memory between iterations of the main M-TIP iteration, and so do not require any communication after initialization. Therefore, apart from the initial scattering operation, these computations are embarrassingly parallel.

**FIGURE 10:**
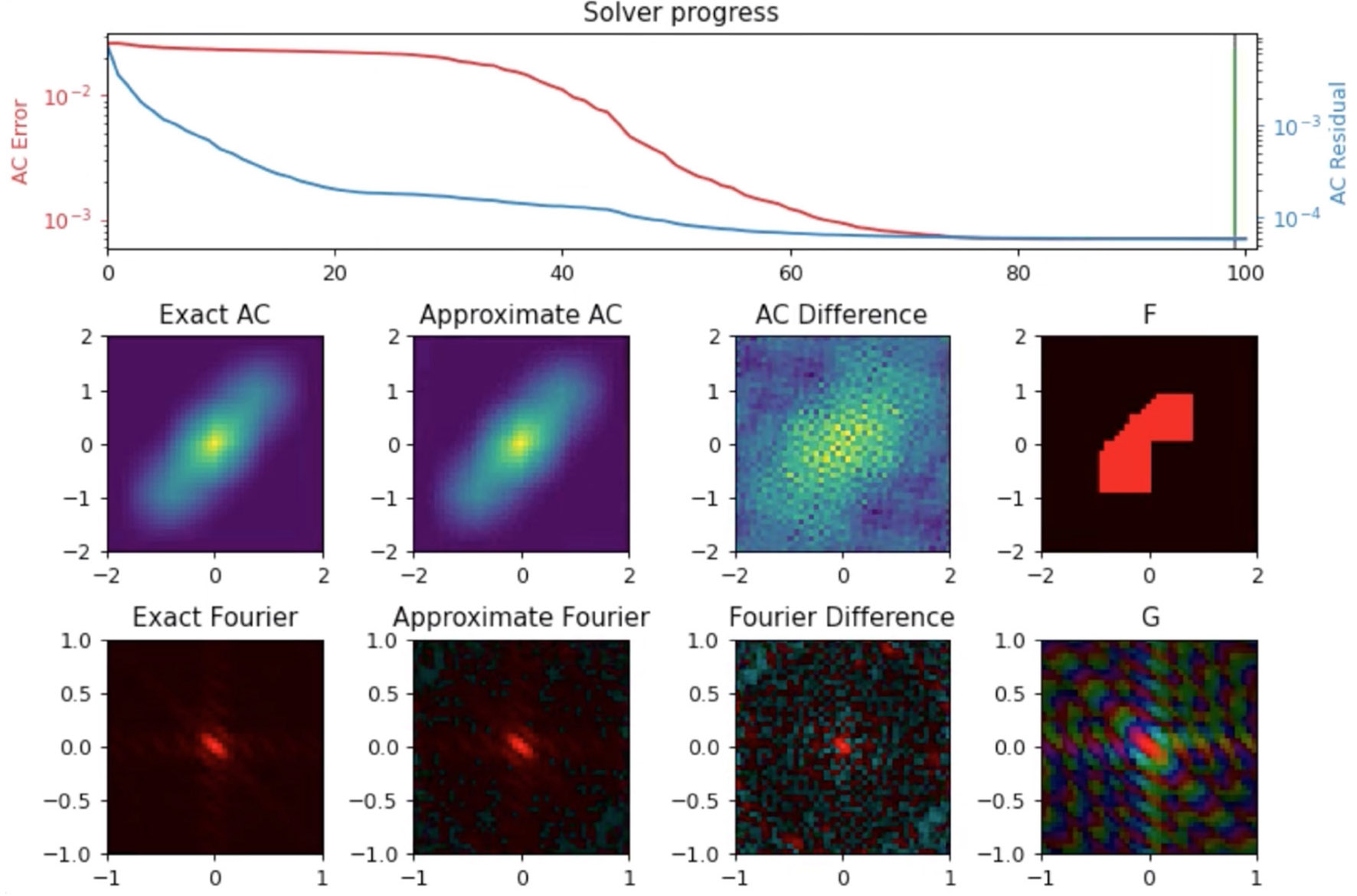
Autocorrelation recovery from NUFFT of model electron density (F); Comparison between the known autocorrelation of the electron density (Exact AC) versus recovered autocorrelation (Approximate AC) show good convergence. G is the Fourier transform of F, the exact AC is given by *G*^2^.

Merging can be formulated as inverting a type-2 NUFFT (uniform to nonuniform grid) on the oriented experimental data to solve for the autocorrelation of the electron density estimate (Figure 10). In particular, the normal equations for this linear system can be expressed as a convolution on a Cartesian grid with dimensions equal to the number of resolution elements per dimension (Fessler et al., 2005). Setting up these normal equations requires two type-1 NUFFTs (nonuniform to uniform grid) to be computed, one on the nonuniform experimental data and one with the nonuniform data replaced with 1’s. With this setup, only the type-1 NUFFT computations (which are computed just one time) need to be distributed, since they are a function of the entire experimental dataset. Fortunately, we can exploit linearity of the NUFFT calculations by computing them separately on the experimental data belonging to each node, and then use a reduction operation to compute their sum. Once these type-1 NUFFTs are computed, the linear system can then be solved efficiently via conjugate gradient, where we can use Cartesian FFTs to efficiently compute the convolution operator in the normal equations. Since these Cartesian FFTs are applied on a grid with size about the same as used for the density model, they can, in principle, be computed on a single node. However, instead of allowing nodes to become idle, we repeat this linear solve over several different nodes, where each node attempts the solve with a different set of hyper-parameters, *e.g*., Tikhonov regularization parameters. Out of these computations with different hyper-parameters, the best solution (in terms of fit to the data and norm of the solution) is then selected.

Phasing does not require access to the experimental data, and only requires computing model values on a Cartesian grid that is about the same size as the density model we wish to reconstruct. Therefore, this step can also, in principle, be efficiently solved on just a single node. However, as was done in the conjugate gradient solve in merging, instead of allowing nodes to become idle, we perform this step over multiple nodes, where different hyper-parameters are tried on each node. The global SPMTIP framework consists of an iterative loop, where during each iteration each of the above three subproblems are solved one after the other. These iterations are repeated until the molecular density model converges to a solution consistent with the data. Additionally, to initialize the process in the first iteration, random orientations are initially given to the different diffraction patterns and the three sub-steps are performed as a first pass. The obtained model is then used as a reference to align the orientations on the second pass. Afterward, the three sub-steps are iteratively applied as described above.

#### 2.3.2 Agile experiments with programming models

ExaFEL took the approach of implementing *Spinifel* with multiple programming model back-ends. It is a holistic approach and forward looking with some R&D aspects. This approach was taken also to mitigate risk for delivering a programmatic version of *Spinifel*; ECP desired more production-ready codes. Multiple programming models had the advantage of providing a seasoned bulk-synchronous programming model or an emerging task-based model. This dual back-end provided the ability to do performance checks and comparisons between both models, as well as comparisons of ease of use and portability. This also enabled research within both an established programming model (MPI) and a task-based programming model (Legion).

**FIGURE 11:**
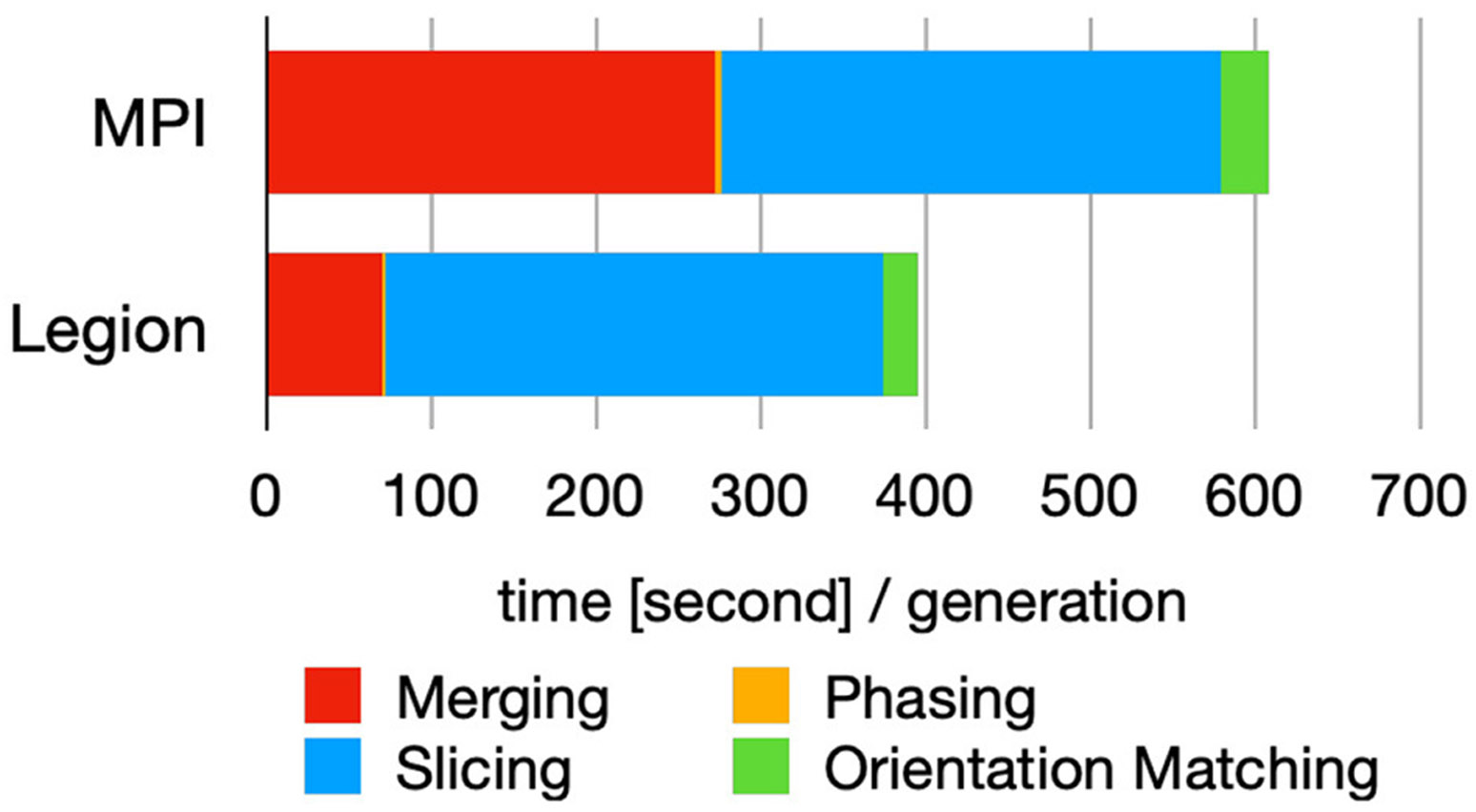
Breakdown of the time spent in each module (*cf.* Figure 9) for each iterative cycle (generation) using MPI and Legion programming model. Figure reproduced from Chang et al. (2021) with the author’s permission.

Figure 11 demonstrates that Legion’s task-based parallelism approach yields improved performance over MPI due to dynamic load-balancing (Chang et al., 2021). In the future, it would be beneficial to explore this further by exercising the different code paths of *Spinifel* and compare programming models within the design space and with different datasets. It is one of the few scalable analytics codes that has the capability to support such research.

#### 2.3.3 Development strategy for portable GPU kernels

Earlier *Spinifel* code and dependencies were designed based on generally portable CPU code and NVIDIA GPU code. While there were no meaningful differences for the CPU code, porting CUDA-related modules, *i.e*., CUDA kernel code for orientation matching, NUFFT, and Python library dependencies like *Numba*, *CuPy*, and *PyCUDA* is non-trivial because they span multiple modules and libraries across both custom code and the broader Python ecosystem, made worse by how the ecosystem was designed and supported by AMD and Intel. In 2023, we completed the port of the *Spinifel* software suite to AMD GPUs. Building on the previous year’s efforts in developing portability layers to various Python ecosystem libraries (*cuFINUFFT, CuPy*, and *PybindGPU*), the last mile involved implementing page-locked memory to support the existing CUDA code, support for Python ecosystem GPU array interfaces, eliminating a dependency on *Numba* which was originally available on AMD GPUs, but then dropped, and vendor-portable, real-time GPU resource tracking. These efforts have resulted in a *Spinifel* code base that runs on both NVIDIA and AMD hardware.

#### 2.3.4 *Spinifel* performance scaling

**FIGURE 12:**
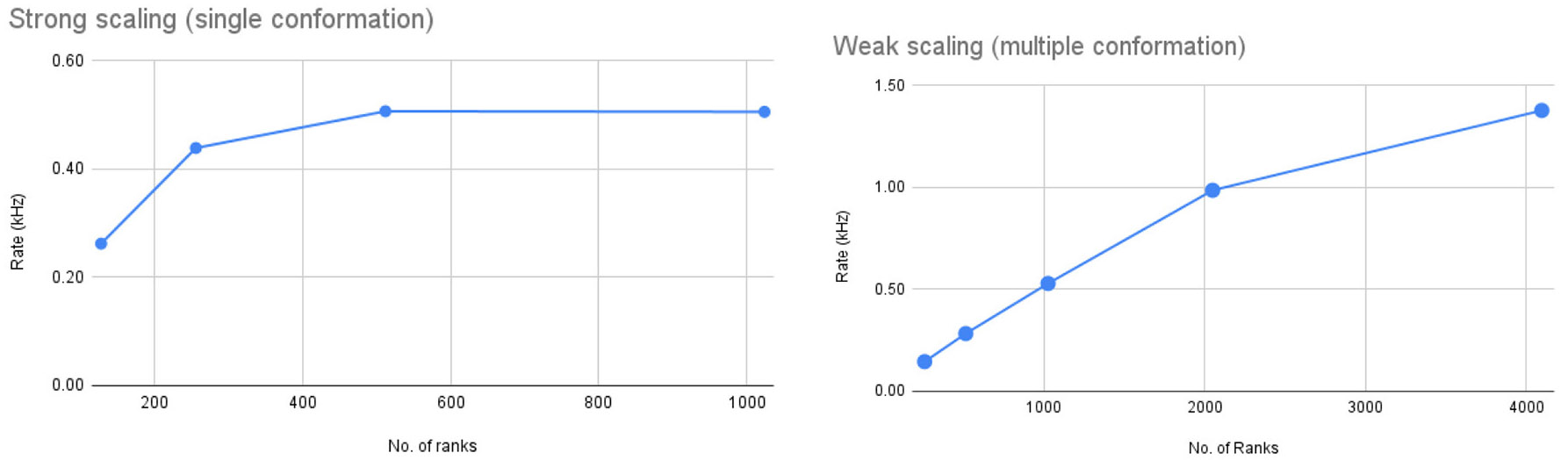
**Left:** Strong-scaling tests running *Spinifel* on Frontier with 131K total images. **Right:** Weak scaling plot running *Spinifel* Legion with simulated diffraction images with two conformations of group II chaperonin. Each rank receives 256 images and is set to run on a fixed 20 generations.

After porting *Spinifel* to Frontier we ran strong scaling tests to determine how well 3D reconstruction with *Spinifel* could keep up with experimental data rates. The test was done on 128–1024 ranks (16–128 nodes with 8 ranks per node setting). The total number of images processed for all the runs is 131,072 (1,024 images per rank for 128 ranks, 512 images per rank for 256 ranks, and so on) with 10 fixed generations. Figure 12 (left) shows the data rate for the four runs. Our scaling runs indicate that for 131 K images, strong scaling stops at approx 512 ranks (or 256 images per rank). Adding more ranks (we tested up to 1,024 ranks) does not result in an overall speedup. During our strong scaling tests, *Spinifel* encounters a runtime error at 128 ranks (16 nodes). We are still investigating this issue.

Group II chaperonin occurs in two possible conformational states: (i) the Open State (PDBID: 3IYF); and (ii) the Closed State (PDBID: 3J03). Real-world sampled often contain a mixture of multiple conformational states. As a simple test of such a situation, we simulated an SPI dataset using thse two conformations. The total number of images used was 1 milion at 128 × 128 pixel resolution. We performed weak scaling by assigning 256 images per rank running from 256 to 4096 ranks (equivalent to 32 to 512 nodes). This test was limited to 512 nodes due to an issue found with the HPE “slingshot” network on Frontier. Each test was set to run with 20 generations. The timing shown in Figure 12 (right) is the total time after start-up until the results are written out. *Spinifel* autocorrelation and phasing phases require all-to-one and one-to- all broadcast operations. As a result, we see a reduction in speedup as we scale to a larger number of processors.

**FIGURE 13:**
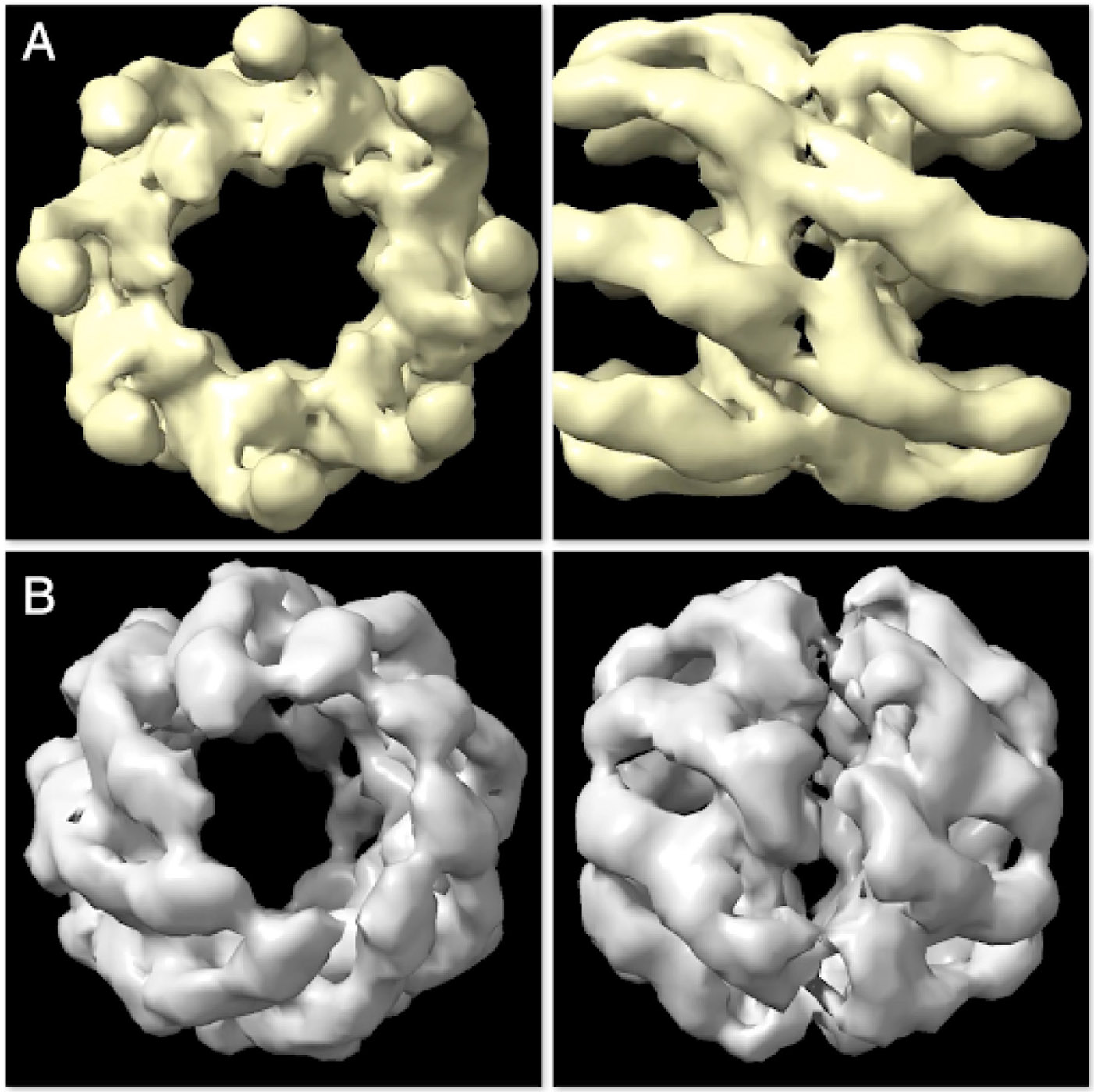
The two constructed structures at generation 20 are found to resemble the Open State **(A)** and Closed State **(B)** of group II chaperonin—demonstrating that *Spinifel* is capable of accurately reconstructing a molecular structure even if the sample contains a mixuture of conformations.

The scaling tests are further evaluated by examining the results of the reconstructed structures. In this case, we expect to see two different models per completion at each generation. Figure 13 shows the reconstructed models at generation 20. The model in Figure 13A (top and side view) resembles the Open State whereas the model in Figure 13B resembles the Closed State of the group II chaperonin.

### 2.4 Python as a scientific HPC workflow language

The majority of ExaFEL’s software products are not singular applications, but rather constitute a suite of tools that collectively generate sophisticated XFEL data analysis workflows using Python as a workflow language. HPC capabilities are leveraged using specialized packages, such as *mpi4py, CuPy, Legion*, and custom Python extension modules, that are compiled using each HPC HPC system’s preferred SDK. In addition to SFX and SPI-specific python extension modules, the ExaFEL project developed the following general purpose HPC python packages which can be used independently from the ExaFEL software suite.

#### 2.4.1 PybindGPU

Having developed kernels for each of the hardware architectures, we lacked a single unified API capable of calling GPU code and managing GPU resources across different vendors. Hence we developed the “PybindGPU” Python module (https://github.com/JBlaschke/PybindGPU) to fill this need. This module provides a Python frontend that unifies the different vendor-specific APIs. This module is built using *Pybind11* and provides:

1. A *NumPy*-compatible array interface using *NumPy*’s buffer protocol;
2. Control over memory placement, *e.g*., GPU data are made available via the __cuda_array_interface__, and pagelocking;
3. Real-time GPU resource tracking for benchmarking. PybindGPU uses CUDA syntax, as the *Spinifel* developers are most familiar with CUDA, and can therefore interface with packages like CuPy even when running on AMD hardware.

#### 2.4.2 Skopi

The *Skopi* (Peck et al., 2022) SPI diffraction simulation package was designed to simulate more realistic experimental conditions in six ways: (1) introducing the ability to place multiple particles at the interaction point, (2) including the ability to simulate self-amplified spontaneous emission (SASE) spectra and beam fluctuation, (3) taking into account the contribution of a hydration layer, (4) expanding the dynamic range of the recorded intensity with the use of autoranging detectors, (5) adding the ability to simulate SPI diffraction patterns of a protein at various conformations, and (6) simulating diverse types of realistic noise such as Poisson noise, beam miscentering, fluence jitter, and static sloped background. We optimize and accelerate the code using GPU-enabled Python packages such as *Numba* and *CuPy*.

#### 2.4.3 FFTX and cuFINUFFT

*FFTX* is the exascale follow-on to the *FFTW* open-source package for executing the discrete Fast Fourier Transform, as well as higher-level operations composed of FFTs and linear operators. It is based on *SPIRAL* (Franchetti et al., 2018), a build-time code generator that produces high-performance kernels targeted to specific uses and platform environments.

Compared to *CuPy*, we found that *FFTX* produced an overall performance boost ranging from 3.85x to 4.95x. Detailed information on our how *FFTX* improves the FFT performance in *Spinifel* is described in Section 2 of the Supplementary material.

Furthermore, only a few Python packages existed for Non-Uniform FFTs. Therefore, the ExaFEL project worked with the *cuFINUFFT* developers (Shih et al., 2021) to make it compatible with *Spinifel*. This involved: (i) enabling multi-GPU support, and (ii) developing a HIP version (as the original code was written in CUDA).

## 3 Real-time data processing for XFEL science

As discussed in Section 1, leveraging exascale HPC resources for real-time data processing necessitates several preparatory activities: already stretching (i) the development of cross-facility workflows (Giannakou et al., 2021; Blaschke et al., 2023, 2024), (ii) the development of codes targeting GPU accelerators (Wittwer et al., 2023; Shah et al., 2024), (iii) the development of infrastructure and services to facilitate cross-facility workflows within HPC data centers (Bard et al., 2022), and (iv) the development of inter-institutional policies and best practices to accommodate workflows bridging experimental facilities and HPC data centers (Miller et al., 2023).

All of these activities are featured in the ExaFEL project. The first two were discussed earlier in Section 2.4. In this section we will focus on the last two.

### 3.1 Facility services for real-time data processing and Integrated Research Infrastructure (IRI)

Broadly speaking, real-time and cross-facility HPC workflows have three ingredients: (i) HPC resources such as compute nodes, (ii) a data plane consisting of high-performance file systems and networking, and (iii) a control plane which allows for workflow tasks to be orchestrated (Antypas et al., 2021). HPC data-centers have traditionally focused on providing the former two (nodes, networks, and file systems), whereas the latter often is a new addition to HPC data-center offerings.

Our experiences with real-time data processing underscore the importance of facilities offering services required by the workflow orchestration control-plane. For ExaFEL these services are described below.

#### 3.1.1 Hosting for persistent state and services

While analyzing data during a live experiment, many things happen between execution of individual compute jobs. These include data transfer, decision logic on which jobs to submit next, and the creation of metadata pertaining to multiple data sets. For ExaFEL this is accomplished by three programs: (i) *XRootD* transfers data from LCLS to the HPC data center, (ii) *cctbx.xfel*’s workflow manager is constantly running in the background, monitoring the state of any file transfers, compute jobs, and user inputs, and (iii) a *MySQL* database stores the state and metadata of any data analysis jobs.

It is therefore crucial that data centers provide appropriate infrastructure host these programs. What makes hosting infrastructure *appropriate* depends on the program being hosted. We identify four conditions needed to effectively orchestrate real-time data analysis workflows: (i) orchestration programs must be able to run unattended longer than the data analysis jobs they manage (usually several days), (ii) data transfer orchestration programs must have simultaneous connectivity to both HPC file systems and external hosts, and iii) workflow orchestration programs must have access to the HPC center’s resource manager (e.g., *Slurm*). Nodes in an HPC system that meet these requirements are collectively known as “Workflow Enablement Nodes.”

We observe that Data Transfer Nodes and Login Nodes often fill this role due to their access to HPC resources and connectivity beyond the data center. However, cloud-native service platforms such as Kubernetes clusters are often more suitable locations due to their flexible and configurable networking, their ability to dynamically scale workloads, and the fact that the micro-services architecture has become a *de facto* standard for hosting many web-services such as APIs and databases.

**FIGURE 14:**
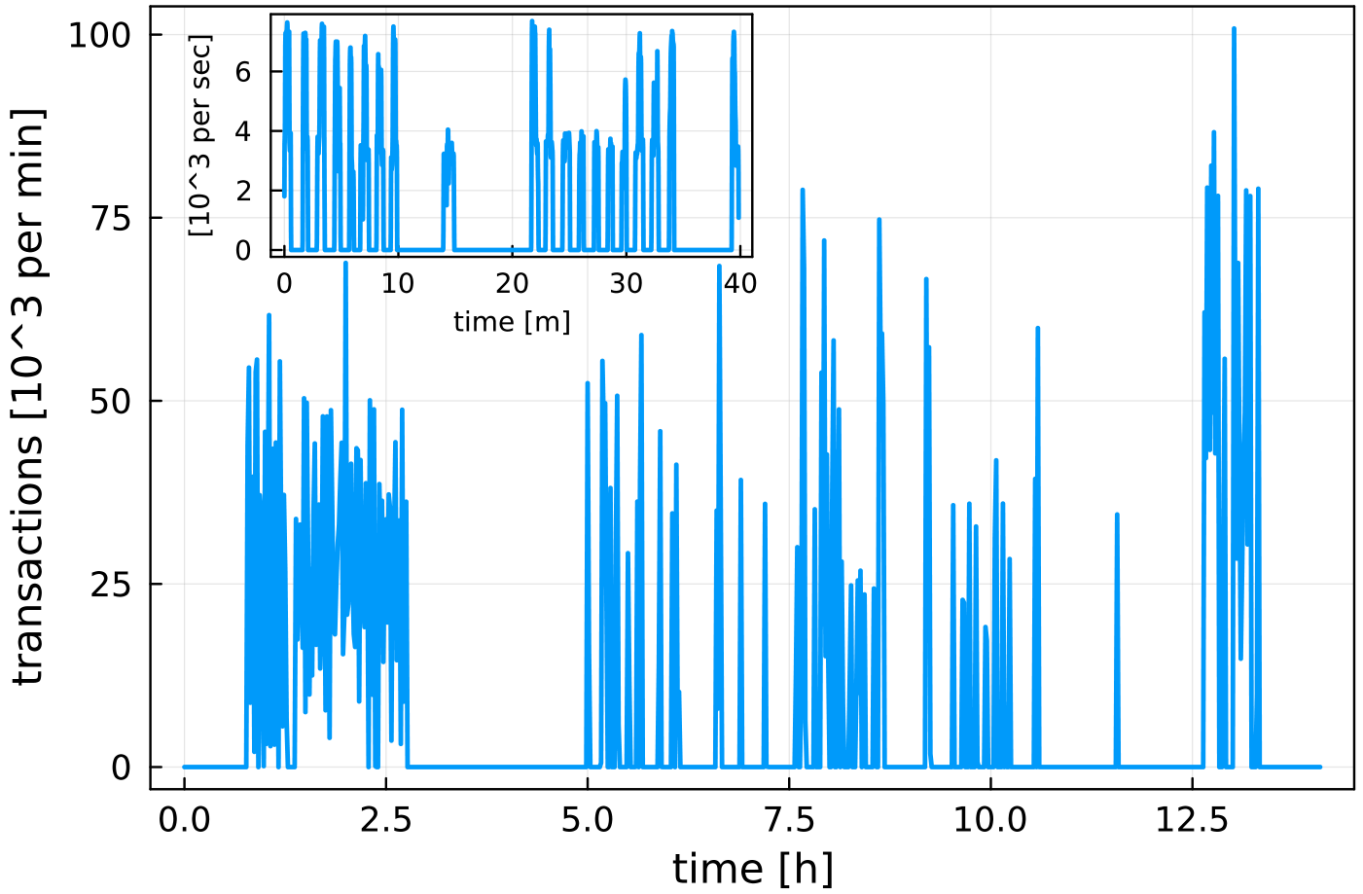
Rate of database transactions during live data processing. The main plot shows the number (in thousands) of database transactions per minute during a 12-h shift. The inset shows a 40-min snapshot of number (in thousands) per second. We see that the database receives up to 8,000 commits/s when data processing takes place; the “bursts” in the inset show individual data analysis jobs. The Spin micro-services platform was capable of handling this heavy load level. Figure reproduced from Blaschke et al. (2024) with the permission of the authors.

By this analysis, NERSC’s Kubernetes-based Spin cluster would be be the preferred location to host *XRootD, cctbx.xfel*, and *MySQL*. However, as Spin does not have access to the scratch file system on Perlmutter, and as additional development work is necessary to access *Slurm* from Spin (via the Superfacility API), at present only *MySQL* is hosted on Spin. *XRootD* runs on dedicated Workflow Nodes using *scrontab*, and *cctbx.xfel* runs on Login Nodes. Figure 14 shows the rate of transactions from 4096 MPI Ranks processed by *MySQL*. This demonstrates that Spin is capable of hosting database services for ExaFEL’s HPC jobs. Current work on Perlmutter and future NERSC systems includes exploring the intergration of platform file systems (such as scratch) and the HPC resource manager (Slurm) with Spin, or similar k8s-based micro-service platforms. Such integration needs to be done carefully to avoid potentially destabilizing Spin (e.g. would a scratch degradation also degrade Spin?). However this work indicates that the payoff is significant, as workflow orchestration, file transfer, and database services could then be autmatically and reliably managed by the same micro-services platform.

#### 3.1.2 Real-time responsive HPC resources

**FIGURE 15:**
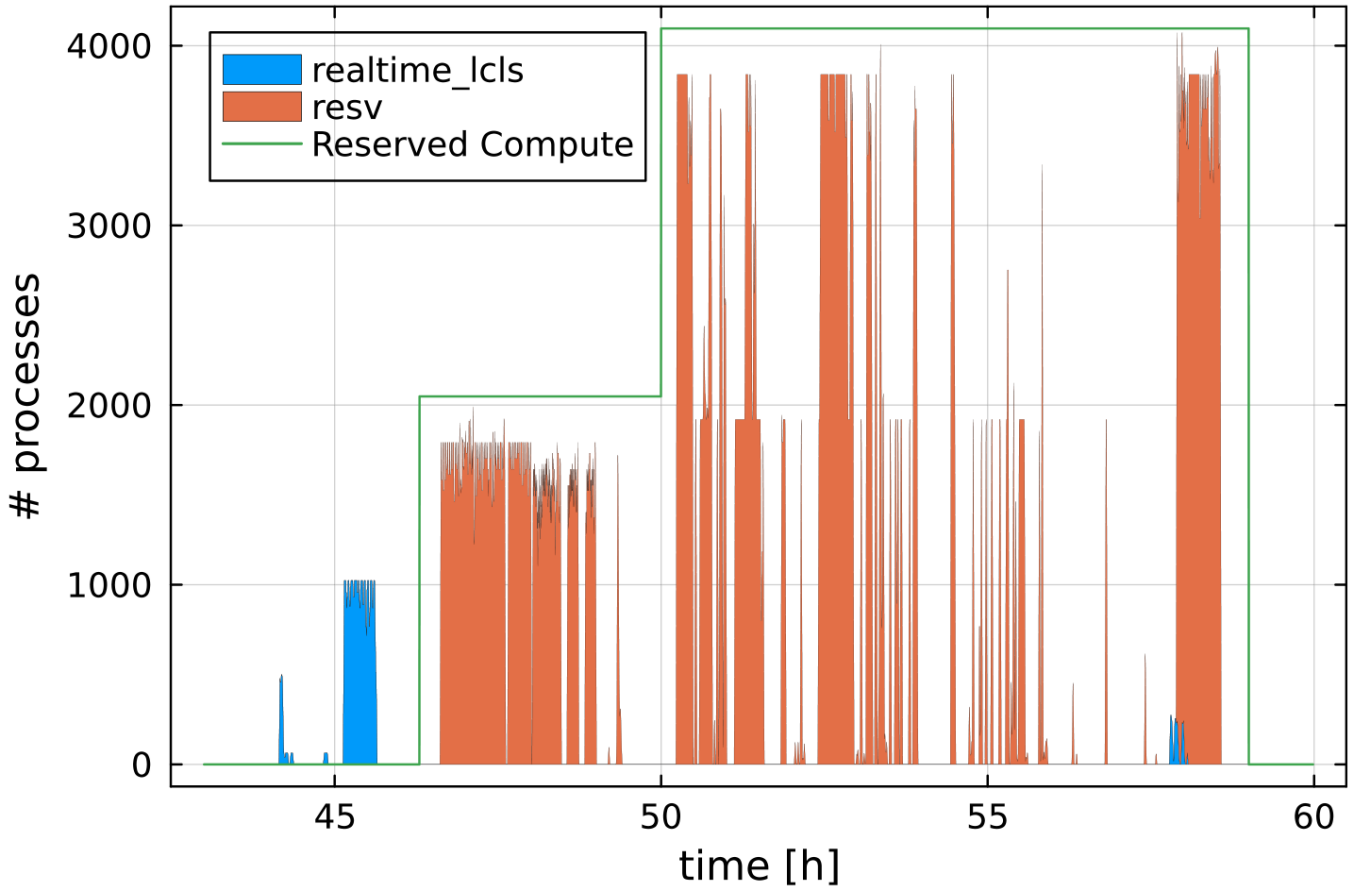
Based on data from Blaschke et al. (2024). Example of the CPU utilization during a 12-h beam-time shift. The “bursty” CPU utilization results from urgent computing tasks: Whenever new data are available they need to be analyzed as quickly as possible. Colors indicate which real-time resource was utilized. The green line shows the number of CPU cores reserved (at 50 hours, the reservation was increased to 64 nodes).

Highly responsive HPC resources are critical components of real-time feedback computing infrastructure. NERSC provides two mechanisms for access to such resources: (i) a small number of nodes (up to 20) are permanently set aside in a “realtime” QOS, reserved exclusively for providing real-time feedback at experiments, and (ii) users can request additional reservations of a variable number of nodes. Figure 15 shows both being used to analyze data at the P175 ^4^ experiment at LCLS (Blaschke et al., 2024). Before the start of the dedicated reservation, preparatory work was carried out using the “realtime” QOS (blue bars). Once the reservation started (green line), live data processing was done using the reserved nodes. The reservation was increased to 64 nodes mid-experiment when it became clear that the existing reservation was too conservative an estimate of the required resources, and the “realtime” QOS was used in addition as overflow. During this experiment, approximately 90% of data processing utilized the reservation, while 10% utilized the “realtime” or the “regular” QOS, depending on urgency.

While exact numbers vary from experiment to experiment, a reservation large enough to accommodate the typical “bursty” use of compute resources will also allow nodes to sit idle at other times, so that only a fraction of allocated resources are ultimately consumed. For example, during the P175 experiment, only about 22% of the reserved node time was utilized. Furthermore, dataset sizes grow over the course of data collection (Blaschke et al., 2024). This phenomenon results in a “triangular” computational burden, with the final stretch of data collection consuming substantially more node hours than earlier segments of the same length. The aforementioned increased reservation during P175, shown by the green line in Figure 15, demonstrates this challenge.

The following overall picture emerges: real-time data processing requires a relatively large amount of HPC resources (compute nodes, file systems, and networks) during relatively short, unpredictable “bursts”. Normally these bursts also become larger over time and are predictable at most a few hours ahead of time.

In order to balance adequate resource availability during bursts with minimization of node-hours spent idle, we have begun exploring *Slurm’s* preemptible reservations. Job preemption ^5^ is a feature offered by the *Slurm* scheduler whereby jobs can be marked as “preemptible” by sending the scheduler a signal to this effect along with a “preemption time,” or minimum time before the job can be checkpointed and stopped to allow other traffic through the queue. In other words, a non-preemptible job can request a node currently running a preemptible job, and the preemptible job will be notified and given a time limit to shut down gracefully before being killed.

**FIGURE 16:**
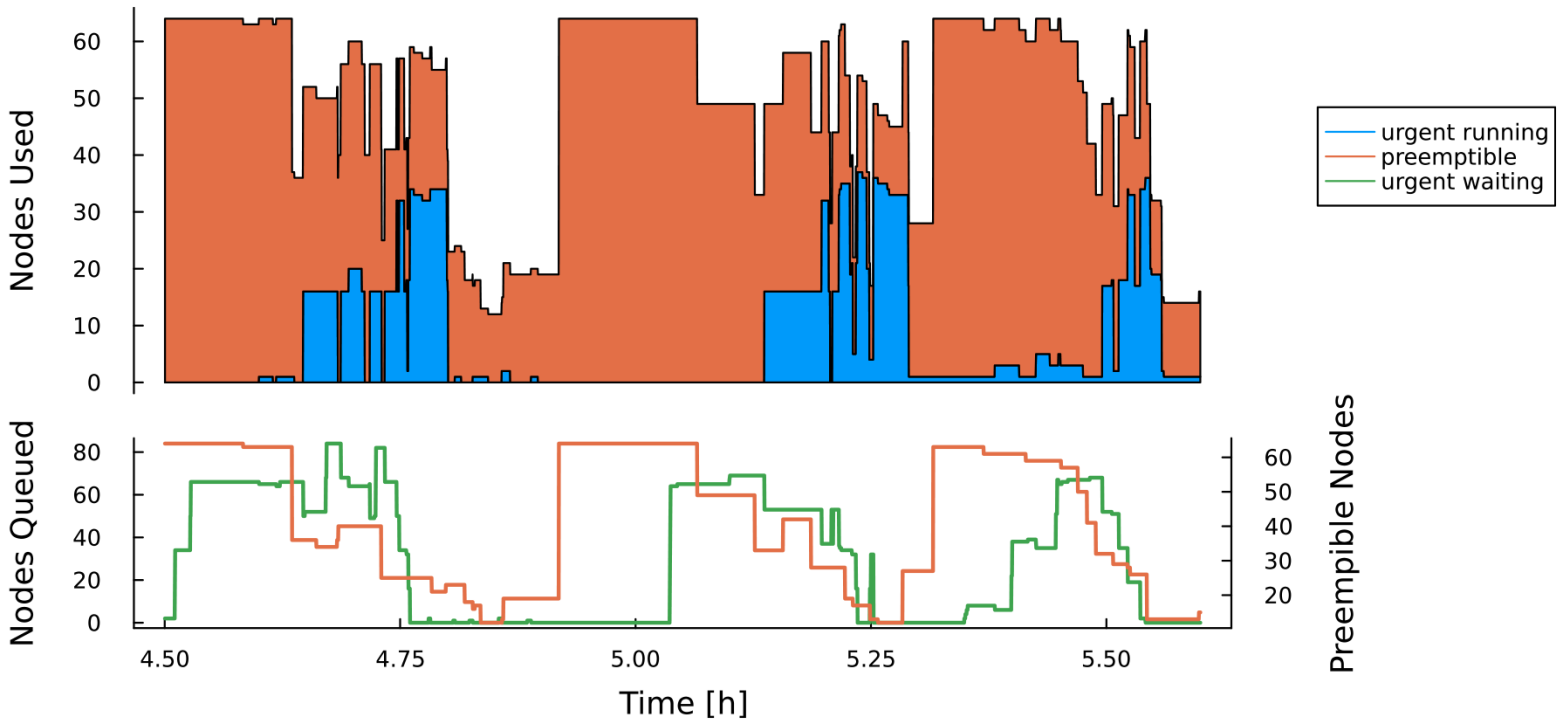
Based on data from Blaschke et al. (2023). Experiment demonstrating preemptible (orange) and urgent (blue) *Slurm* jobs sharing a reservation. **Top:** preemptible jobs successfully fill any gaps between urgent jobs. **Bottom:** *Slurm*’s job preemption system in action: when urgent jobs enter the reservation (green line), preemptible jobs are given a warning signal to exit gracefully, and then are killed 5 minutes later (orange line). All-in-all this does delay urgent jobs but improves overall utilization.

This mechanism extends to reservations as well: preemptible jobs may use idle nodes in a reservation, provided the preemption time is less than a specified setting. In effect, the longest preemption time of any preemptible jobs in a given reservation is the upper limit on wait time for a non-preemptible job to start. In Figure 16 we show the results of an experiment testing the delay incurred by allowing preemptible jobs access to a reservation. Under those conditions, jobs belonging to the reservation used approximately 12% of the reservation’s total node-hours, while preemptible jobs used approximately 65%.

**FIGURE 17:**
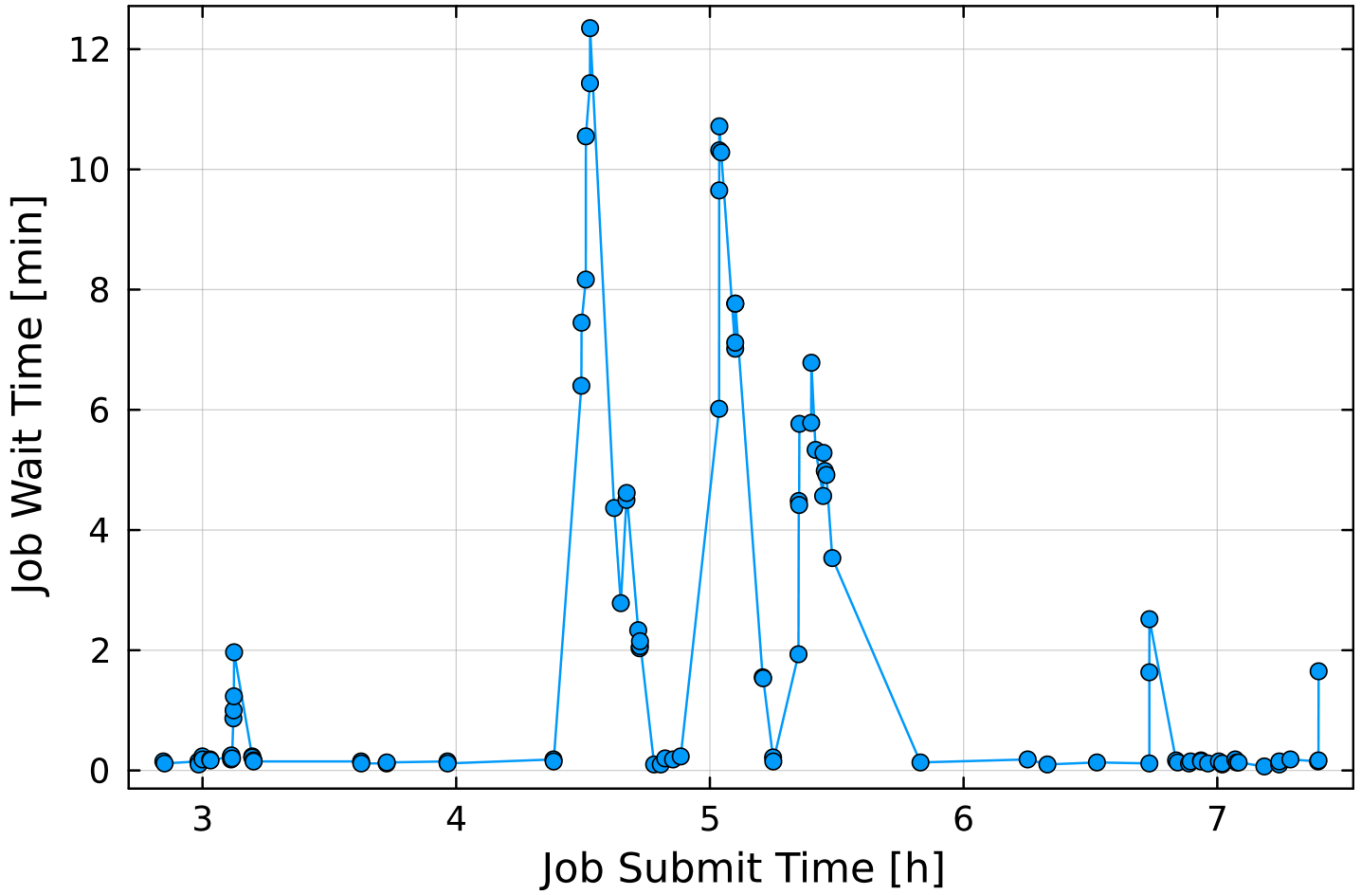
Job-startup delay as a function of time for the preemptible reservation shown in Figure 16. The “spikes” in job wait time between hour 4 and 6 coincide with multiple urgent jobs being submitted to the reservation. As *Slurm* has to give jobs up to the full preemption time (in this experiment a preemption time of 5 min was chosen), several jobs had to wait for over 10 min.

Allowing preemption increases the overall utilization of reserved nodes (in the above case to approximately 77%), but at the cost of significant startup delays while waiting for preemptible jobs to shut down. Figure 17 shows job wait time as a function of submit time. In this example, most preemptible jobs consumed the full 5 min allowed before shutting down, resulting in wait times of over 10 min for some backlogged, non-preemptible jobs. To date, we have not found a method to accurately predict maximum wait time in a preemptible reservation.

#### 3.1.3 Collaborative user environments

Data analysis from ExaFEL experiments is a highly collaborative process: raw data from instruments and analysis artifacts need to be accessible to the whole team driving the experiment. Our experience has been that default Unix file permissions often hamstring this effort by preventing others from editing shared files, and sharing login credentials is not an acceptable workaround.

At NERSC, the ExaFEL team has access to two valuable tools that facilitate collaborative data analysis: (i) collaboration accounts, named *collabsu*, and (ii) shared database services. Collaboration accounts are managed using NERSC’s *Iris* ^6^ account management platform. Project PIs (and designated PI proxies) can manage collaboration accounts (e.g., by adding or removing users) internal to their projects, and users can run the *collabsu* shell command to switch their user environment to the collaboration account. Shared database services likewise allow users to manage access permissions.

Together these services create a shared user environment to which multiple team members have access. As a standard practice, all collected data and analysis artifacts are accessible within this shared user environment. An unintended but advantageous consequence of this approach is that user settings (e.g., Unix dotfiles) are also shared. This means that all users of the *cctbx.xfel* software via the *collabsu* account use the same settings, greatly accelerating some forms of troubleshooting.

### 3.2 Policies and best practices for integrating experiments and HPC data centers

Many of the services listed in Section 3.1 rely on corresponding institutional policies and best practices in order to function properly. For example, NERSC’s Spin micro-services platform is located within the data center’s network and can accommodate up to 8000 transactions per second (Figure 14), a rate made possible by that network’s low latency. Also, NERSC users can quickly reserve compute nodes (within a week of submitting a reservation request). Both of these capabilities are the result of institutional policy. The former results from a networking security policy that allows arbitrary micro-services access to the same network as the compute nodes, and the latter depends on a policy to permit reservations for as long as users have a justifiable need.^7^

Facility services listed in Section 3.1 serve to streamline resource management and cross-institutional collaboration. It is therefore important for HPC centers and IRI partner facilities to develop and maintain a best practice of assessing whether center policy is disruptive to cross-facility workflows, and if so, how to minimize the disruption. Using Spin as an example, preventing micro-services platforms from running arbitrary Docker images would appear to be a good policy from a security perspective, but it would severely limit the kinds of workflows that can run at scale. NERSC’s Spin takes a permissive approach to micro-services within the data center network, provided users have undergone the necessary training and the micro-services pass security checks enforced by Spin’s Open Policy Agent (OPA).^8^

## 4 Experiences and lessons learned while developing XFEL data analysis on HPC systems

Here we detail our experiences running ExaFEL on NERSC’s Perlmutter and OLCF’s Frontier systems.

Each Perlmutter GPU compute node consists of 1x 64-core AMD EPYC 7763 with access to 256 GB of DDR4 RAM. Each node also contains 4x NVIDIA A100 GPUs. Each Frontier compute node consists of 1x 64-core AMD EPYC 7453 CPU with access to 512 GB of DDR4 RAM. Each node also contains 4x AMD MI250X, each with 2 Graphics Compute Dies (GCDs), for a total of 8 GCDs per node.

### 4.1 HPC workflow management and visualization

Initial efforts prioritized simple command-line scripts to process data, alongside other scripts to dynamically write the batch submission script. Keeping track of file paths and experiment metadata rapidly became tedious at best, and at worst critically delayed real-time feedback. To this end, a HPC-capable workflow manager was designed and integrated into *cctbx.xfel* to streamline the process and enable real-time feedback. The workflow manager consists of two components: an interactive graphical user interface (GUI) that generates *Slurm* job scripts and manages job dependencies, and a *MySQL* database backend that tracks the progress of compute jobs in real time (*cf*. Sections 3.1.1). An intentional design element of the workflow manager is that the generated jobscripts are retained, allowing jobs to be archived for debugging purposes.

#### 4.1.1 Performance visualization

**FIGURE 18:**
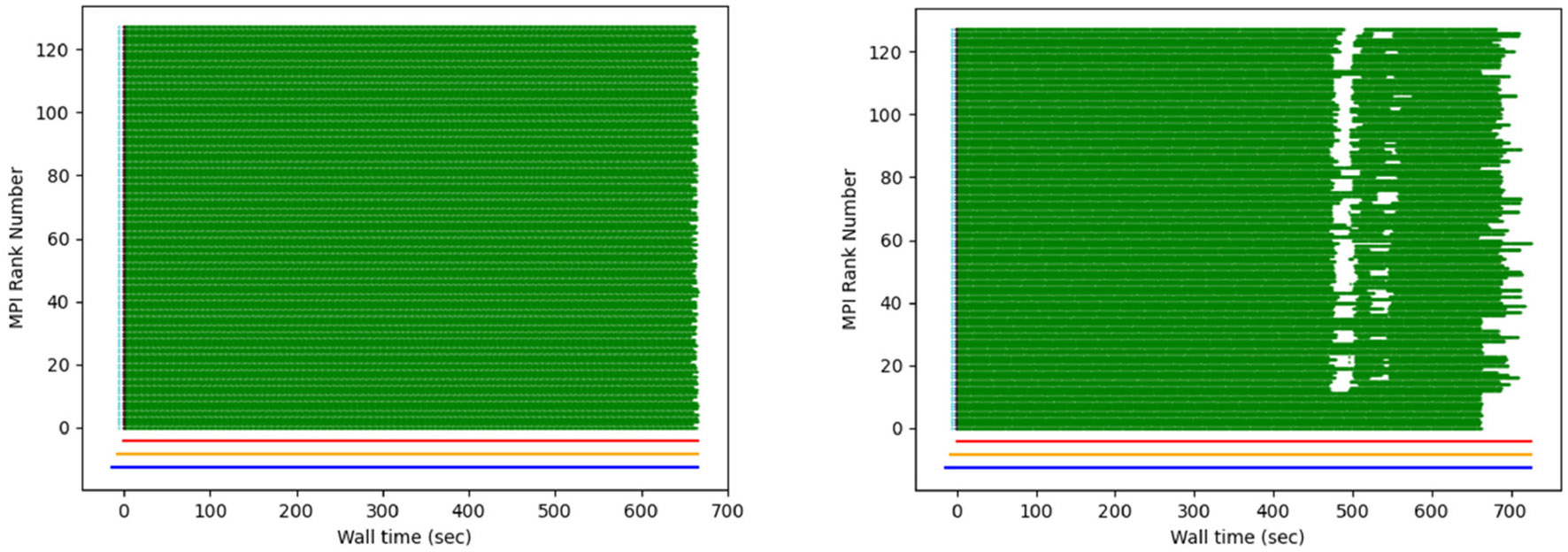
Example of a “weather plot”: each MPI rank is assigned a different row. When a task is completed, a green dot is plotted at the corresponding wall time. The figure on the right shows the effect of intermittent I/O contention. Figure reproduced from Blaschke et al. (2023) with the author’s permission.

One of the first XFEL experiments that included data transfer and processing at NERSC was hindered due to slow loading of Python modules on compute nodes. As hundreds of MPI ranks loaded the same Python source files, the filesystem lagged, leading to long start-up times. This was ultimately solved by using OCI-compatible containers, but initially a lack of information precluded rapid diagnosis of the problem. To this end, the main processing script was modified to (optionally) create debug files that report the time of completion for each processing step of each diffraction pattern. From these, we developed the “computational weather plot” (Figure 18). These plots are discussed extensively in Blaschke et al. (2024), but briefly, they display the processing rate for every rank. At a glance they reveal I/O bottlenecks, nodes with poor network access, metadata synchronization issues, and problematic images. They have been used to solve a variety of problems, both online (during beam-times) and offline.

### 4.2 I/O lessons learned for HPC scientific data analysis applications

With full diffraction datasets including as many as 10^7^ diffraction patterns, there is a potential “small file problem” if the data reduction results from each diffraction pattern are written to individual files, in which case the large number of metadata operations would quickly overwhelm the filesystem. This problem is compounded by the fact that we generate intermediate results at each step of the analysis pipeline, Figure 5a-e, and even for each sub-step of the *dials.stills_process* step (Figure 5a) as detailed in Section 2.2.1. For this reason we provide the (default) option of serializing the intermediate results in composite containers, in different formats (json, Python pickle, and pandas dataframe) depending on the analysis step. In this design we write out one container file per MPI worker rank, so that Figure 6 would result in 10 output files, exclusive of the rank 0 controller. When needed, each step of our pipeline has access to the original raw data on flash memory (such as the performance tier of the Orion filesystem of Frontier), either in HDF5 or *psana/xtc* format, thus eliminating any need to create and store big data (such as corrected images) to temporary files.

**FIGURE 19:**
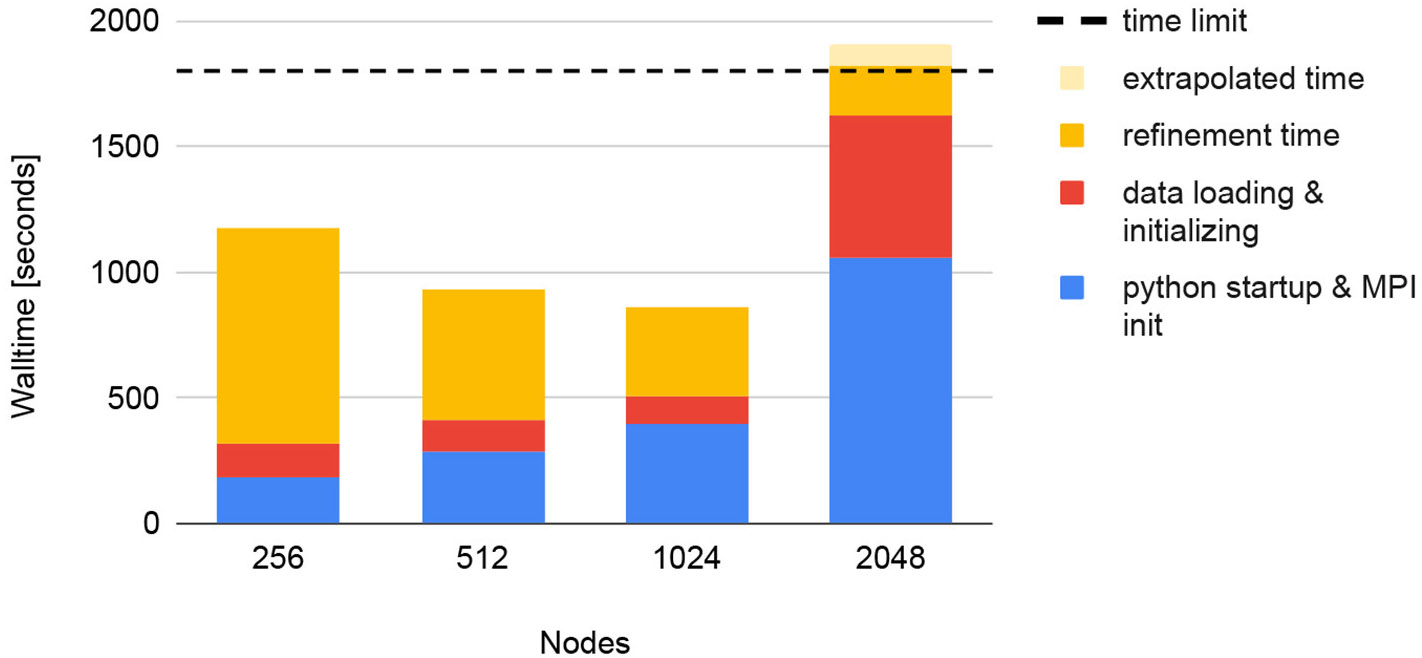
Strong scaling tests of *diffBragg stage 2* on Frontier for reconstructing 130,000 images. Using more nodes leads to faster refinement but longer start-up times. At some point, this becomes unsustainable, resulting in the job exceeding its time limit (horizontal dashed line).

We tested the scaling of all I/O operations used by *diffBragg*. For this test, both the data and the program installation were stored on Frontier’s “Orion” high-performance scratch filesystem. Keeping all reconstruction parameters constant, we reconstructed the dataset with four different node configurations, using 256, 512, 1,024, and 2,048 Frontier nodes. Figure 19 summarizes the results. Up to 1,024 nodes, the overall runtime goes down, but it increases abruptly when 2,048 nodes are used. This is the result of two simultaneous effects. The first is a faster refinement time on a larger number of nodes: increasing the number of nodes from 256 to 1,024 reduces the refinement time from 850 s to 350 s. For 2,048 nodes, the refinement could only finish 312 of the 451 iterations within the 30-min time limit. However, because the time per iteration is constant, we can extrapolate that it would take 290 s to complete 451 iterations. This speed-up is offset by longer startup times with increasing node count. For 256 nodes, it takes 190 s to start Python and initialize MPI. This grows to 400 seconds for 1,024 nodes, already longer than the refinement time of 350 seconds. For 2,048 nodes, the startup time grows to over 16 min, longer than the complete runtime for the 512 node run.

To reduce these long startup times, we implemented the mitigation described in Section 4.3 below.

### 4.3 Importance of the runtime environment to Python applications

Python imports require searching and linking libraries in PYTHONPATH. As reported in Giannakou et al. (2021), Python startup times therefore scale very poorly on shared file systems. At the start of the project, to run on multiple nodes, we deployed OCI-compatible container technology such as *Docker, Shifter*, and *Podman-HPC*. The container runtime caches the image’s contents (and therefore all Python modules) on node-local storage, making it accessible optimally on the compute nodes. The container approach was implemented as part of the previous NERSC system (Cori) in 2019 using *Shifter*, and later implemented on Perlmutter in 2022 using *Podman-HPC*. As of FY23, OLCF Frontier does not support container technology. Investigating Python import for *Spinifel* and all its dependencies shows that it takes ~5 minutes on 100 nodes and >30 minutes on 1000 or more nodes. This presents a problem for ExaFEL, which aims to achieve SPI data processing rates of 5 kHz. Figure 19 shows similar Python load time scaling for *cctbx*.

To approximate the functionality of a container runtime, we packed the entire application environment (Python code, compiled code, and all dependencies) into a tarball on Frontier’s Lustre filesystem, which was then broadcast at run time (using sbcast) to flash memory on each node. The observed start-up time with this fix is shown in Table 2. However, code changes are now more time consuming and complicated, as each change requires extracting the tar archive, applying the changes, and then creating an updated tar archive. Therefore, it must be stressed that this approach is at best a workaround.

## 5 Conclusion

ExaFEL is a HPC-capable XFEL data analysis software suite for both Serial Femtosecond Crystallography (SFX) and Single Particle Imaging (SPI) developed in collaboration with the Linac Coherent Lightsource (LCLS), Lawrence Berkeley National Laboratory (LBNL) and Los Alamos National Laboratory. ExaFEL supports real-time data analysis via a cross-facility workflow spanning LCLS and HPC centers including NERSC and OLCF. Our work constitutes initial path-finding for the US Department of Energy’s (DOE) Integrated Research Infrastructure (IRI) program.

In this work, we discussed the ExaFEL team’s 7-years experience and results in developing XFEL data analysis code for the DOE Exascale machines. Furthermore we reviewed essential data center services (and their implications on institutional policy) required for real-time data analysis. We summarized our software and performance engineering approaches and our experiences with NERSC’s Perlmutter and OLCF’s Frontier systems. Our efforts have resulted in an exascale-ready software suite capable of running real-time data analysis workflows on modern supercomputers, as well as the body of knowledge and experience necessary to maintain and further develop this workflow for upcoming experiments.

We expect that the ExaFEL workflow will be an essential data analysis tool for real-time data processing during LCLS-II’s future high data-rate experiments. Furthermore, we hope this review is a practical blueprint for similar efforts in integrating exascale compute resources into other cross-facility workflows.

## Supplementary Material

Supplementary material

The Supplementary Material for this article can be found online at: https://www.frontiersin.org/articles/10.3389/fhpcp.2024.1414569/full#supplementary-material

## Figures and Tables

**TABLE 1:** Performance of *nanoBragg* to simulate 100,000 diffraction images.

Host system	Hardware per node	Time	Nodes	MPI ranks
Edison	2 × Intel Xeon E5-2695v2	12.3 h	280	6,720
Cori/KNL	1 × Intel Xeon Phi 7250	7.2 h	484	8,228
Summit/CUDA	6 × NVIDIA V100	91 s	297	12,474
Perlmutter/CUDA	4 × NVIDIA A100	297 s	80	1,280
Perlmutter/Kokkos	4 × NVIDIA A100	251 s	80	640

Each test used 5% of the system nodes. The number of MPI ranks was determined to best hide latency on each system.

**TABLE 2:** Startup times for *Spinifel* from the compressed (and sbcast’ed) Python environment on Frontier.

No. of nodes	100	1,000	2,000	4,000
sbcast (s)	60	80	73	84
Python import (s)	6.85	51.34	67.47	175.85
exiting (s)	0.15	4.66	4.53	8.15
Total time (s)	67	136	145	268

## Data Availability

The raw data supporting the conclusions of this article will be made available by the authors, without undue reservation.

## Footnotes

1
https://www.es.net/

2
https://www.boost.org/doc/libs/1_84_0/libs/python/doc/html/index.html

3
https://github.com/pybind/pybind11

4 “P175” is an identifier used by LCLS to describe a particular allocation of beamtime.

5
https://slurm.schedmd.com/preempt.html

6
https://iris.nersc.gov

7
https://docs.nersc.gov/jobs/reservations/

8
https://docs.nersc.gov/services/spin/
