# Supplementary material for: ExaFEL: extreme-scale real-time data processing for X-ray free electron laser science
Source: Front High Perform Comput. Author manuscript; Available in PMC 2025 Oct 30. (PMC12570208; doi:10.3389/fhpcp.2024.1414569)
Supplement: Supplementary material [file NIHMS2113343-supplement-Supplementary_material.pdf]

Supplementary Material

1 DETAILED DESCRIPTION OF THE COMPUTATIONAL CRYSTALLOGRAPHY TOOLBOX

The computational crystallographic toolbox (*cctbx*) was introduced over 20 years ago as a collaborative open-source software library. It provides a foundation of core algorithms, expressing the mathematics of crystals, including unit cells, space groups, and crystal diffraction (Grosse-Kunstleve et al., 2002). *cctbx* uses Python for programmatic ease, coupled with *Boost.Python* to underlying C++ code to afford speed and performance. *cctbx* forms the basis for several crystallographic packages for the two general steps of diffraction data processing.

DIALS (Diffraction Integration for Advanced Light Sources, Winter et al. (2018)) is one package that is built on the *cctbx* library, giving an open source implementation of specialized data reduction algorithms. *DIALS* provides robust support for images in a variety of file formats from light-sources as diverse as the smallest labs to the largest facilities.

The *cctbx.xfel* (Brewster et al., 2019) toolkit uses *DIALS* to perform the specific task of processing images from XFELs. *cctbx.xfel* can analyze images up to 10 kHz in real time, producing reports and giving fast feedback that scientists can use to direct experiments. The data reduction steps performed by *DIALS* are enumerated here:

1. Import: the image is read. Metadata from the header and user-supplied parameters are gathered to construct the beam and detector models, giving context to the raw pixel data.
2. Spotfinding: the pixel data are searched for regions of connected bright pixels that arise from Bragg diffraction, usually 2-20 pixels, depending on the detector type.
3. Indexing: the spots from the detector are mapped onto reciprocal space to do a search for crystal parameters (unit cell dimensions) that best fit the observed spots.
4. Refinement: optimization methods are used to refine experimental parameters to best fit the observed data.
5. Integration: the experimental models (beam, detector, and crystal) are used to predict which reflections should be visible on the detector, including weak signal (near background level) arising from weak structure factors or high-angle reflections that are too faint for visible contrast.
6. Correction and filtering: reflection intensities are corrected for polarization arising from the configuration of the instrument, and reflections belonging to crystals with invalid or poorly measured unit cells are excluded from further analysis.
7. Scaling: each image is scaled to a reference dataset to account for shot-to-shot variation in beam energy and crystal size/quality.
8. Post-refinement: reflection intensities are refined once again, this time adjusting crystal orientation, disorder parameters, and the per-image scale factor to minimize differences between the reflection measurements and the reference intensities. This includes determining a partiality correction, which accounts for the Bragg reflections which come from still images (no crystal rotation) being not quite in the diffraction condition.

The *diffBragg* toolkit (Mendez et al., 2020) within *cctbx* is intended to address a trade-off that degrades the accuracy of XFEL data in comparison to conventional measurements at synchrotron sources. While XFELs offer superior time resolution down to a few femtoseconds, the X-ray spectrum and intensity is fluctuating from pulse to pulse. This is in contrast to synchrotron sources, which are typically monochromatic and stable up to a linear scale factor. At synchrotrons, the crystal orientation and Bragg reflection profiles are carefully controlled using a goniometer. At XFELs, the variations between diffraction patterns creates a need for each pixel intensity to be corrected by an individual scaling factor that accounts for influences such as the incident X-ray spectrum and the internal crystal disorder. *diffBragg* implements a new algorithm to determine these scaling factors using an inverse modeling approach: rather than calculating the structure factors by simply summing the pixel intensities within a box around a Bragg spot, *diffBragg* uses a physics model of the experiment to predict the size, shape, and intensity profile of each spot, adjusting its parameters, including the structure factor amplitudes, to most closely fit the observed data. This detailed modelling requires GPU-based parallelism, so that the analysis can be performed on the same time scale as the experiment .

2 FFT PERFORMANCE OPTIMIZATIONS USING FFTX

The "phasing" operation in *Spinifel* takes as inputs two real 3D arrays, density ρ and amplitude A, and modifies the Fourier-transformed density $\hat{\rho}$ replacing its magnitude with the amplitude A, while keeping the phase unchanged. The result is then transformed back to the spatial domain. The Python code for the phasing kernel in *Spinifel* is shown in Fig. S1.

```
rho_hat_ = cupy.fft.fftn(rho_)
phases_ = cupy.angle(rho_hat_)
amp_mask_ = cupy.ones((M, M, M), dtype=cupy.bool_)
amp_mask_[0, 0, 0] = 0
rho_hat_mod_ = cupy.where(amp_mask_,
    amplitudes_ * cupy.exp(1j*phases_), rho_hat_)
rho_mod_ = cupy.fft.ifftn(rho_hat_mod_).real
```

Figure S1. Python code in *Spinifel* for phasing, where input arrays are `rho_` and `amplitudes_`, both of dimensions `M` × `M` × `M`. Output array is `rho_mod_`, also of dimensions `M` × `M` × `M`.

The code segment shown in Fig. S1 can be replaced by a call to an *FFTX* function:

```
import fftx
rho_mod_ = fftx.convo.stepphase(rho_, amplitudes_)
```

By optimizing the phasing kernel as a whole, *FFTX* is able to reduce communication and improve performance. We ran *Spinifel* on Frontier on arrays of size $M \times M \times M$, where M is fixed within each run. For each run with a different setting of M, Table S1 shows the number of calls to the phasing kernel, the maximum relative difference between the two results, and the speedup of the *FFTX* function over the original kernel with *CuPy*, as measured by the ratio in mean times after the first call.

The maximum relative differences shown in Table S1 are all close enough to double-precision roundoff error, and the speedup of *FFTX* over the kernel with *CuPy* for phasing is from 3.85x to 4.95x.

Table S1. *Spinifel* phasing kernel using FFTX

M	number of calls	maximum relative error	FFTX speedup
81	6500	5.27e-13	4.00x
105	6500	3.54e-13	3.85x
125	6500	3.23e-13	4.12x
165	6500	2.09e-13	4.92x
189	6500	2.69e-13	4.95x
225	6500	2.02e-13	4.35x

The "free-space convolution" operation in *Spinifel* takes as inputs two real 3D arrays, φ of dimensions $M \times M \times M$, and G of dimensions $2M \times 2M \times 2M$, and computes the following:

$$(G * \varphi)(\boldsymbol{j}) \equiv \sum_{\boldsymbol{k} \in [0,\ldots,M-1]^3} G(M\boldsymbol{u} + \boldsymbol{j} - \boldsymbol{k})\varphi(\boldsymbol{k}) \tag{S1}$$

for $\boldsymbol{j} \in [0, \ldots, M-1]^3$, where $\boldsymbol{u} = (1, 1, 1)$.

This can be calculated efficiently using the discrete 3D Fourier transforms of size $2M \times 2M \times 2M$. The Python code for free-space convolution in *Spinifel* is shown in Fig. S2.

```
ugrid_ups = cupy.zeros((2*M,)*3, dtype=uvect.dtype)
ugrid_ups[:M, :M, :M] = ugrid
F_ugrid_ups = cupy.fft.fftn(
    cupy.fft.ifftshift(ugrid_ups))
F_ugrid_conv_out_ups = F_ugrid_ups * F_ugrid_conv_
ugrid_conv_out_ups =
    cupy.fft.fftshift(cupy.fft.ifftn(F_ugrid_conv_out_ups))
ugrid_conv_out = ugrid_conv_out_ups[:M, :M, :M]
```

Figure S2. Python code in *Spinifel* for free-space convolution, where the input arrays are `ugrid`, corresponding to φ in Equation (S1), of dimensions $\mathtt{M} \times \mathtt{M} \times \mathtt{M}$, and `F_ugrid_conv_`, corresponding to $\hat{G}$, of dimensions $\mathtt{2M} \times \mathtt{2M} \times \mathtt{2M}$. Output array is `ugrid_conv_out`, of dimensions $\mathtt{M} \times \mathtt{M} \times \mathtt{M}$.

The code segment shown in Fig. S2 can be replaced by a call to an *FFTX* function:

```
import fftx
ugrid_conv_out = fftx.convo.mdrconv(ugrid, F_ugrid_conv_)
```

As with the phasing kernel, by optimizing the free-space convolution kernel as a whole, *FFTX* is able to reduce communication and improve performance. For each run of *Spinifel* with a different setting of M, Table S2 shows the number of calls to the free-space convolution kernel, the maximum relative difference between the two results, and the speedup of the *FFTX* function over the original kernel with *CuPy*, as measured by the ratio in mean times after the first call.

The maximum relative differences in Table S2 are all close enough to double-precision roundoff error, and the speedup of *FFTX* over the kernel with *CuPy* for free-space convolution is from 4.04x to 4.62x.

Table S2. *Spinifel* free-space kernel using FFTX

M	number of calls	maximum relative error	FFTX speedup
81	481	1.06e-15	4.17x
105	481	9.31e-16	4.62x
125	481	8.60e-16	4.11x
165	468	1.09e-15	4.59x
189	467	1.04e-15	4.56x
225	457	1.11e-15	4.04x

REFERENCES

Brewster, A. S., Young, I., Lyubimov, A., Bhowmick, A., and Sauter, N. K. (2019). Processing serial crystallographic data from XFELs or synchrotrons using the cctbx.xfel GUI. *Computational Crystallography Newsletter* 10, 22–39

Grosse-Kunstleve, R. W., Sauter, N. K., Moriarty, N. W., and Adams, P. D. (2002). The *Computational Crystallography Toolbox*: crystallographic algorithms in a reusable software framework. *Journal of Applied Crystallography* 35, 126–136. doi:10.1107/S0021889801017824

Mendez, D., Bolotovsky, R., Bhowmick, A., Brewster, A. S., Kern, J., Yano, J., et al. (2020). Beyond integration: modeling every pixel to obtain better structure factors from stills. *IUCrJ* 7, 1151–1167. doi:10.1107/S2052252520013007

Winter, G., Waterman, D. G., Parkhurst, J. M., Brewster, A. S., Gildea, R. J., Gerstel, M., et al. (2018). DIALS: implementation and evaluation of a new integration package. *Acta Crystallogr D Struct Biol* 74, 85–97. doi:10.1107/s2059798317017235
